# The E-cadherin/AmotL2 complex organizes actin filaments required for epithelial hexagonal packing and blastocyst hatching

**DOI:** 10.1038/s41598-017-10102-w

**Published:** 2017-08-25

**Authors:** Sebastian Hildebrand, Sara Hultin, Aravindh Subramani, Sophie Petropoulos, Yuanyuan Zhang, Xiaofang Cao, John Mpindi, Olli Kalloniemi, Staffan Johansson, Arindam Majumdar, Fredrik Lanner, Lars Holmgren

**Affiliations:** 1 Department of Clinical Sciences, Intervention and Technology (CLINTEC), Karolinska Institutet and Division of Obstetrics and Gynecology, Karolinska University Hospital, Huddinge, Sweden; 2 Department of Oncology-Pathology, Cancer Centrum Karolinska (CCK), Karolinska Institutet, Stockholm, Sweden; 3 Department of Medical Biochemistry and Microbiology, Uppsala Biomedical Center (BMC), Uppsala University, Uppsala, Sweden; 4 Medical Biotechnology, VTT Technical Research Centre of Finland, Turku, Finland; 5 Institute for Molecular Medicine Finland (FIMM), University of Helsinki, Helsinki, Finland; 6 Present Address: Eli Lilly and Company, Lilly Corporate Center, Indianapolis, IN 46285 USA

## Abstract

Epithelial cells connect via cell-cell junctions to form sheets of cells with separate cellular compartments. These cellular connections are essential for the generation of cellular forms and shapes consistent with organ function. Tissue modulation is dependent on the fine-tuning of mechanical forces that are transmitted in part through the actin connection to E-cadherin as well as other components in the adherens junctions. In this report we show that p100 amotL2 forms a complex with E-cadherin that associates with radial actin filaments connecting cells over multiple layers. Genetic inactivation or depletion of amotL2 in epithelial cells *in vitro* or zebrafish and mouse *in vivo*, resulted in the loss of contractile actin filaments and perturbed epithelial packing geometry. We further showed that *AMOTL2* mRNA and protein was expressed in the trophectoderm of human and mouse blastocysts. Genetic inactivation of amotL2 did not affect cellular differentiation but blocked hatching of the blastocysts from the zona pellucida. These results were mimicked by treatment with the myosin II inhibitor blebbistatin. We propose that the tension generated by the E-cadherin/AmotL2/actin filaments plays a crucial role in developmental processes such as epithelial geometrical packing as well as generation of forces required for blastocyst hatching.

## Introduction

A central question during development is how single cells form functional multi-cellular organ structures. The high reproducibility indicates intricate synchronization of cellular processes such as migration, proliferation and cell shape changes. Much attention has been focused on how growth factors form biochemical gradients that govern some of these processes^1–3^. However, less is known regarding how mechanical signals or forces modulate cell shape and control cellular expansion^4, 5^.

Cells perceive and respond to exogenous mechanical forces via different points of contact in the outer membrane. Forces exerted on the extra-cellular matrix are detected by epithelial cells via integrins in focal adhesions which transfer tension from the extracellular matrix to the cytoskeleton^6^. Low rigidity in the extra-cellular matrix transfers less extracellular force and thereby promotes the formation of organ-like epithelial structures *in vitro* whereas increased force or stiffness in the matrix causes loss of tissue architecture associated with tumor progression and promotes cell proliferation^7–10^.

Recent evidence has also shown that actomyosin contractility is transmitted via the adherens junctions. External forces applied to cadherins have indicated a mechanical coupling between the cytoplasmic domain of cadherin and the actin cytoskeleton^11^. Cellular interactions and the force-mediated morphological changes are also important for the processes involved in organ development. One example is apical contraction where the apical side of cells contracts to a wedge-like shape required for sheets of cells to fold or bend to form invaginations e.g. during Drosophila germ band extension, vertebrate gastrulation or neural tube formation^12–14^. An important issue is how force is transmitted from E-cadherin to the cytoskeleton. Classical cadherins are normally associated to p120, β and α–catenins, which are essential for the connection to actin filaments. Recent evidence suggests that α-catenin may undergo force-dependent conformational changes that regulate binding of the minimal cadherin-catenin complex to an actin filament under force. Force-induced conformational changes also allows binding of effector proteins such as vinculin dependent on junctional maturity and myosin II activity^15, 16^.

The angiomotin scaffold protein family is comprised of angiomotin (amot), angiomotin like 1 (amotL1) and angiomotin like 2 (amotL2). Each protein exists in two different isoforms, whereat the two amotL2 isoforms are called p100 amotL2 and p60 amotL2. All three amot family members have been studied extensively in endothelial cells, demonstrating their importance in cell migration, polarization, proliferation and tight junction stability^17–20^. Furthermore, the amot family of proteins has been shown to be vital for maintaining polarity, regulating cell growth and motility, and facilitating tight junction stability^21–24^. Amot has been reported to bind F-actin, thereby controlling cell shape in endothelial cells^25^ and facilitating actin cytoskeleton remodeling in epithelial cells^26^. p100 amotL2 has been shown to localize to the cellular junctions of epithelial tissue cells with so far undescribed functional impact^27^. We have previously shown that amotL2 is essential for normal vascular development, specifically during vasculogenesis where amotl2 associates to VE-cadherin to mediate actomyosin-dependent mechanical force required for aortic expansion^28^. Finally amot and amotL2 have further been shown to control lineage specification of the first cell type of the mammalian embryo, the trophectoderm^29, 30^ which also is the first epithelial tissue to form.

In this report, we have analyzed the functional role of amotL2 in epithelial cell-cell junctions in several cultured epithelial cells lines *in vitro* as well as in zebrafish skin epithelium and mammalian trophectoderm *in vivo*. We show that amotL2 is a component of the E-cadherin complex that is essential for the formation of radial actin filaments. Functionally, depletion of amotL2 and subsequent loss of radial actin fibers resulted in stalled hatching of mouse and human embryos from the *zona pellucida*.

## Results

### AmotL2 expression in epithelial cells

Analysis of *AMOTL2* mRNA levels in organ tissues revealed a ubiquitous expression in all organs except lymphoid, blood and bone marrow cells (Supplemental Fig. 1a). Furthermore, amotL2 expression in 755 human cell-lines *in vitro* indicated that amotL2 is primarily expressed in epithelial cells (Supplemental Fig. 1b).

**Figure 1:**
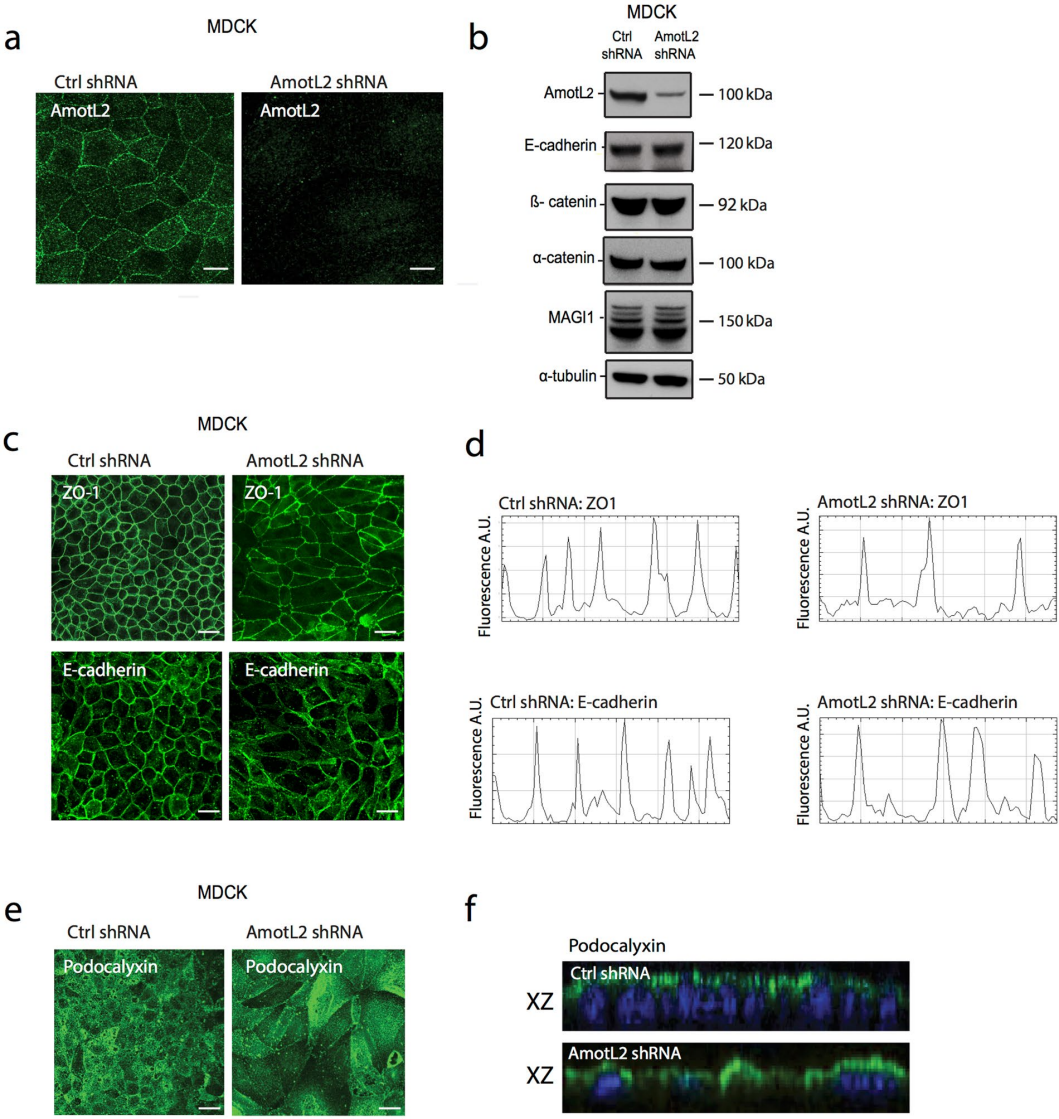
Expression of AmotL2 in epithelial cells and tissues. (**a**) Immunofluorescent staining using affinity purified rabbit polyclonal antibodies generated against the c-terminal domain of amotL2. AmotL2 signal localized to cellular junctions (left panel) and was significantly lowered in MDCK amotL2 shRNA transfected cells (right panel). Scale bars = 10 µm. (**b**) Western blot analysis of steady-state protein levels of amotL2 and junctional proteins in Ctrl shRNA or AmotL2 shRNA transfected MDCK cells. Protein levels of junctional proteins were not affected by amotL2 depletion. (**c**) Immunofluorescent stainings of Ctrl or Amotl2-depleted cells as indicated. Junctional localization of the tight junction marker, ZO1, or cell to cell adhesion marker, E-cadherin, was not affected as shown in the profile plots shown in (**d**). (**e**) Immunofluorescence staining of MDCK cells with apical membrane marker podocalyxin. Scale bars = 10 µm. (**f**) Images of podocalyxin staining in the XY axis showed targeting of podocalyxin to apical membranes in both Ctrl and shAmotL2 MDCK cells. All experiments were performed minimum three times with similar results.

To analyze potential role of amotL2 in formation and maintenance of cell-cell contact, we depleted amotL2 protein levels using shRNA carrying lentiviruses targeting approach as previously described^31, 32^. Three epithelial cell lines were utilized: Madin-Darby Canine Kidney (MDCK) cells, which are tumorigenic kidney epithelium cells derived from dog, Caucasian colon (Caco-2) cells, a human epithelial colorectal adenocinoma cell line and an immortalized human keratinocyte cell line (HaCaT) derived from human skin. The knock-down efficiency was analyzed by immunofluorescent staining and by western blot (Fig. 1a,b and Supplemental Fig. 2a and b). The effect of amotL2 depletion on the junctional localization of tight junction protein ZO-1 and the adherens junction protein E-cadherin was assessed by immunofluorescence staining (Fig. 1c). As shown in the profile plots in Fig. 1d, both proteins still localized to cell-cell junctions. Amot proteins bind to the Par3 and Crb3 apical polarity protein complexes^21, 33, 34^. We therefore assessed whether removal of amotL2 protein would perturb apical-basal polarity. MDCK cells were stained antibodies against the apical protein podocalyxin (Fig. 1e). As shown in Fig. 1f, podocalyxin could be detected in the apical membranes of both Ctrl and amotL2 shRNA MDCK cells.

### p100 amotL2 is required for actin organization

**Figure 2:**
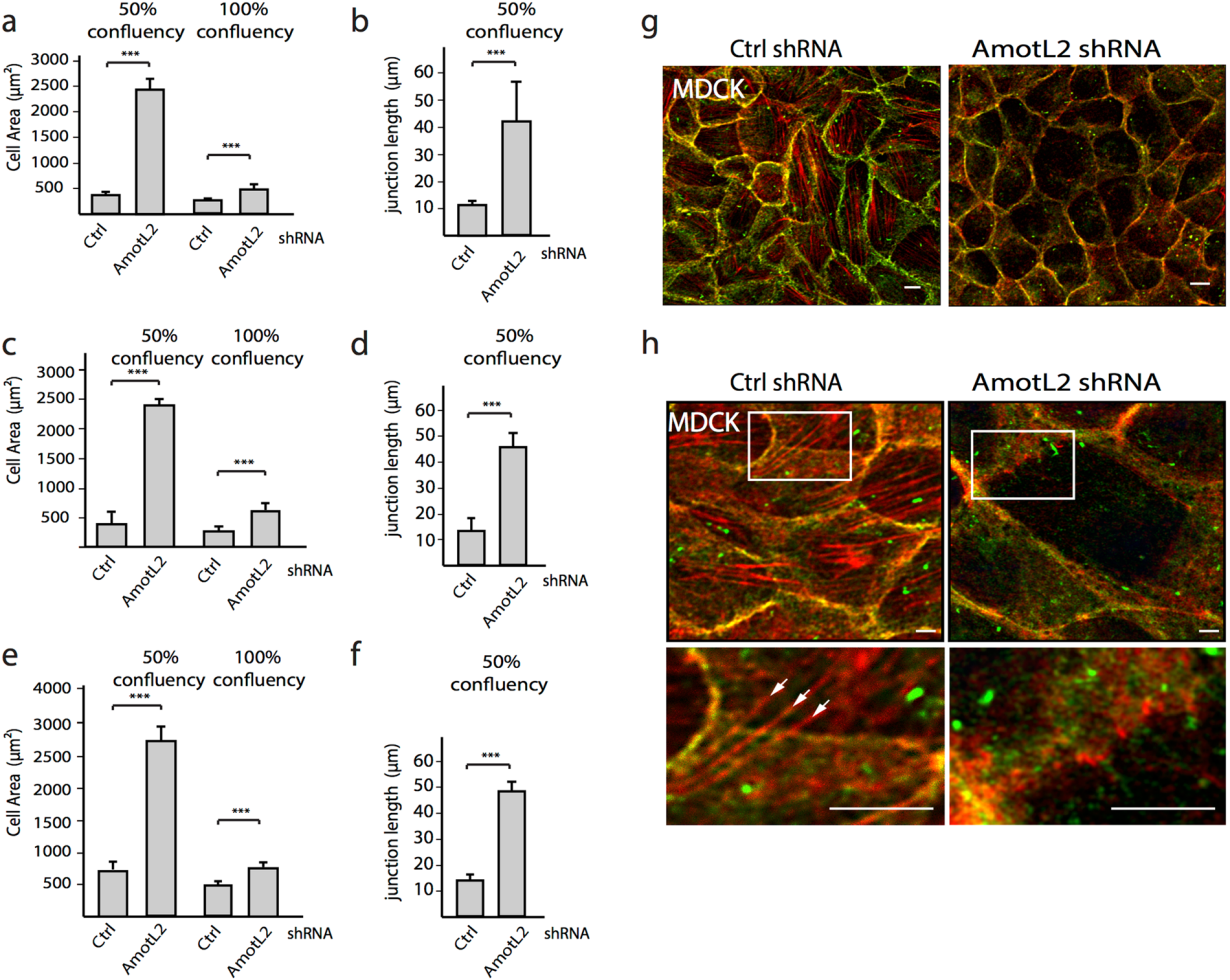
A role of AmotL2 in regulating cell area and actin filament structures. (**a**,**c** and **e**). Bar diagrams show cell area in Ctrl shRNA and AmotL2 shRNA transfected cells at 50% and 100% confluency. (**b**,**d**,**f**) shows quantification of junctional length at 50% confluency. (**a**,**b**) MDCK cells. (**c**,**d**) Caco2. (**e**), (**f)**. HaCat cells. ***p < 0.001. (**g**) Immunofluorescent staining of E-cadherin (green) in MDCK cells as indicated. Actin filaments were visualized using phalloidin staining (red). (**h**) Super-resolution imaging of actin filaments. Note para-cellular actin filaments in the Ctrl shRNA MDCK cells (in frame and in high magnification arrows), which were not detectable in amotL2 shRNA, transfected cells. All experiments were performed minimum three times with similar results. Scale bars = 10 µm.

The amotL2 depleted cells exhibited a markedly altered cellular morphology when cultured at 50% confluency compared to that of the control cells. At 50% confluency MDCK cells grow in loosely packed colonies. The changes in cellular surface area and junction length were quantified and a ~6-fold increase in cell area and a ~4-fold increase in junction length was detected in amotL2 shRNA cells (Fig. 2a–f). The cellular surface area phenotype could be rescued by re-expression of p100 amotL2 (Supplemental Fig. 2c) or was partially restored in densely packed cell cultures (Fig. 2a,c and e).

Next we investigated whether changes in cytoskeletal components could explain the altered cell shape. However, the distribution of F-actin using phalloidin staining was dramatically altered. Typically, control cells exhibited junctional actin as well as fibers that were apparently connecting to filaments of the neighboring cells via membrane junctions (Fig. 2g and h). In contrast, amotL2 shRNA cells typically lacked the radial actin fibers that connected perpendicular to the cell membrane (Fig. 2g,h and Supplemental Fig. 2). The localization of the tubulin or keratin networks were not visibly affected (Supplemental Fig. 3).

**Figure 3:**
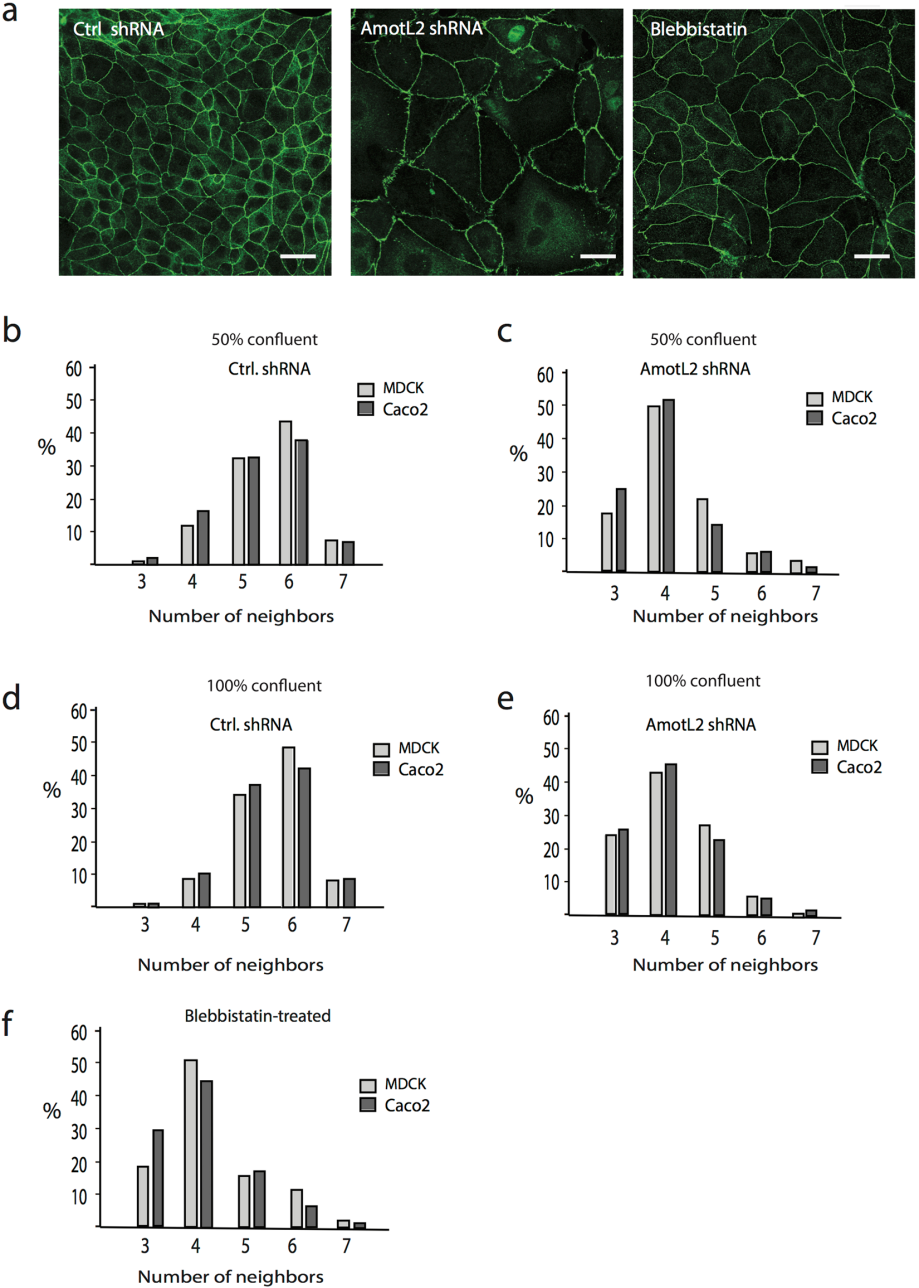
AmotL2 is essential for epithelial geometrical packing. (**a**) Caco2 cells were stained with the ZO1 to visualize cellular outline and geometrical shapes. Scale bars = 25 µm. (**b**,**c**,**d**,**e** and **f**). Quantification of number of cell neighbors in Ctrl shRNA, AmotL2 shRNA or blebbistatin-treated MDCK and Caco2 cells as indicated. The differences in the number of neighbors were statistically analyzed using the two-sample Kolmogorov-Smirnov test. MDCK Ctrl vs. AmotL2 shRNA, p < 0.001; MDCK Ctrl vs. blebbistatin, p < 0.001; Caco2 Ctrl vs. AmotL2 shRNA, p < 0.001; Caco2 Ctrl vs. blebbistatin, p < 0.001. Data are derived from three independent experiments.

The packing of cell layers into hexagonal shapes was originally observed by Lewis in 1928 and is a geometrical form that is universally conserved, extending from plants, sea urchins to human epithelia^35, 36^. ZO1 staining of control or amotL2 shRNA MDCK showed clear differences in surface areas and geometrical shape (Fig. 3a). The number of sides bordering neighboring cells was quantified and their relative frequencies were summarized in the bar diagrams in Fig. 3b and c. Caco-2 and MDCK cells consisted to the great majority of cells with either pentagonal or hexagonal shape (Fig. 3b). Following amotL2 knock-down with shRNA the cell shape was drastically changed with most cells bordering to four neighboring cells (Fig. 3c). Interestingly, densely packing cells did not affect (in contrast to the cell area shown in Fig. 2) the geometrical shape of the epithelial cells (Fig. 2d and e). With the rationale that amotL2 knock-down cells showed deficiency in the microfilament network, we hypothesized that loss of actomyosin contractility could account for the change in cell shape. As such, we treated control cells with the myosin II inhibitor, blebbistatin, before quantifying cell geometry. Myosin II inhibition via blebbistatin treatment has been shown to facilitate actin fiber disassembly^37^. Interestingly, a 2-hour treatment with blebbistatin drastically affected epithelial cell shape, mimicking the effect of amotL2 depletion (Fig. 3a and f). Taken together, we show that amotL2 is required for organizing the actomyosin network to control cell area, size and shape.

### AmotL2 is part of the E-cadherin junctional protein complex

**Figure 4:**
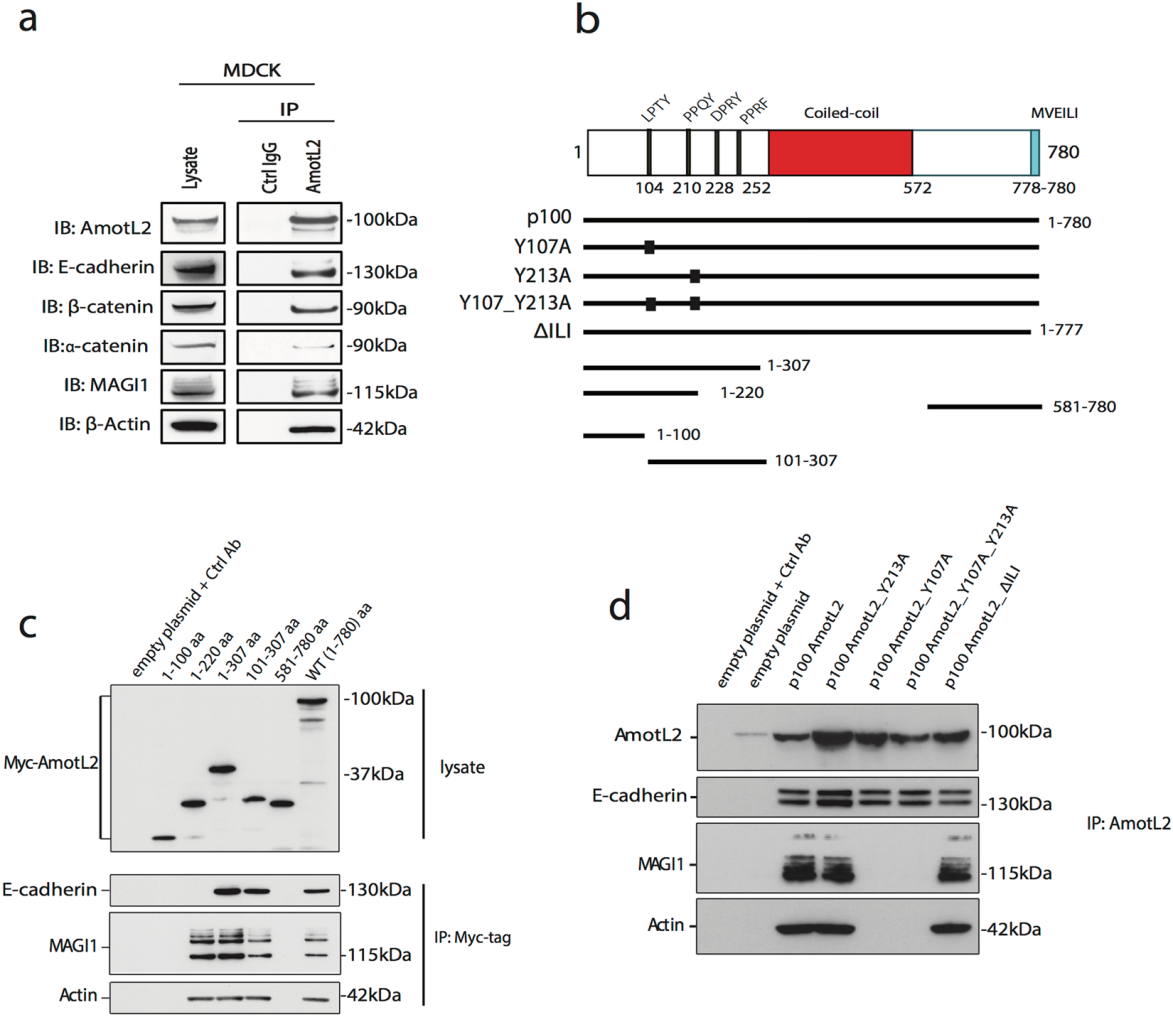
AmotL2 associates to the E-cadherin junctional complex. (**a**) Endogenous amotL2, co-immunoprecipitates with E-cadherin, α-catenin, β-catenin, MAGI1 and actin in MDCK cells. The experiment was repeated four times with the same result. Affinity purified rabbit IgG was used as negative control. (**b**) Schematic model depicting the protein binding domains of the human p100 AmotL2 protein (ensemble ENST00000422605). Putative coiled-coil domain (314–572), C-terminal PDZ-binding motif (778–780) and the N-terminal LPTY (104–107) and PPQY (210–213) WW-binding sites. Listed are also the deletion constructs (black lines) and the mutation constructs used to define the E-cadherin binding site. (**c**). Immunoprecipitation analysis using exogenous transfected fragments of human amotL2 (as shown in (**b**)). Comparison of the binding patterns to the distinct fragments identified a domain between 220–307 a.a. as being essential for the association to E-cadherin. The experiment was repeated three times showing the same result. (**d**) Mutation of the PPQY motif (Y213A), LPTY (Y107A) or deletion of the PDZ-binding motif (ΔILI) did not affect the binding to E-cadherin. When the LPTY motif was mutated to LPTA (Y107A), the binding of AmotL2 to MAGI1 and actin was lost. The double mutant construct LPTA_PPQA (Y107A_Y213A) showed a binding efficiency similar to the LPTA (Y107A) mutant. Thus, these data show that MAGI1/actin and E-cadherin associate to distinct binding sites of the N-terminal domain of AmotL2. The experiment was repeated three times with the same result.

We have previously shown that p100 amotL2 associates to the VE-cadherin complex in endothelial cells^28^. The corresponding cadherin in epithelial cells is E-cadherin which has been extensively studied as loss of protein function or expression has been implicated in tumor progression and invasion^38, 39^. Next we assessed whether amotL2 was associated to the E-cadherin junctional complex in epithelial cells. For this purpose, we performed co-immunoprecipitation analysis and could show that amotL2 was directly or indirectly bound to E-cadherin as well as α and β-catenin (Fig. 4a). In addition, we could further demonstrate that amotL2 is associated with the junctional scaffold protein MAGI1 and actin (Fig. 4a). We and others have shown that the amot family of proteins associates to the scaffold junctional protein MAGI1 via a WW protein interaction motif^27, 28, 40^. It has further been reported that MAGI1 associates to VE-cadherin via binding to β-catenin^41^. These findings raised the possibility that MAGI1 acts as a direct link between actin-amotL2 and E-cadherin. To identify the amotL2 domains responsible for the binding to E-cadherin, we performed co-immunoprecipitation analysis using deletion mutants covering the N-terminal protein interaction motifs as well as the C-terminal PDZ-binding motif (Fig. 4b). Using this strategy, we identified a domain of 87 a.a. which was responsible for the interaction between amotL2 and E-cadherin (Fig. 4c). This domain contains two potential WW-protein interaction sites (Fig. 4b), which were mutated by substituting the tyrosine to an alanine. However, none of these sites appeared to be essential for the association to E-cadherin as analyzed by co-immunoprecipitation (data not shown). The association of MAGI1 and actin was mapped to the 101–220 a.a. N-terminal domain of p100 amotL2 which was distinct from the E-cadherin interaction site. By mutating the tyrosine to alanine we could show that MAGI1 binding was lost in the LPTA mutant (Fig. 4d). In addition, the association of actin to amotL2 was dependent on the LPTY motif (Fig. 4d). All Angiomotin protein family members also contain a C-terminal PDZ-binding motif (Fig. 4b). This motif binds directly to the PATJ/MUPP1 or the Par3 polarity proteins^21, 33, 42^. However, neither the LPTY/PPQY motifs nor the C-terminal PDZ-binding motif was required for the interaction with E-cadherin (Fig. 4d). In conclusion, these data show that E-cadherin MAGI1/actin associate to amotL2 via separate domains.

### AmotL2 sensitizes epithelial layers to external mechanical force

**Figure 5:**
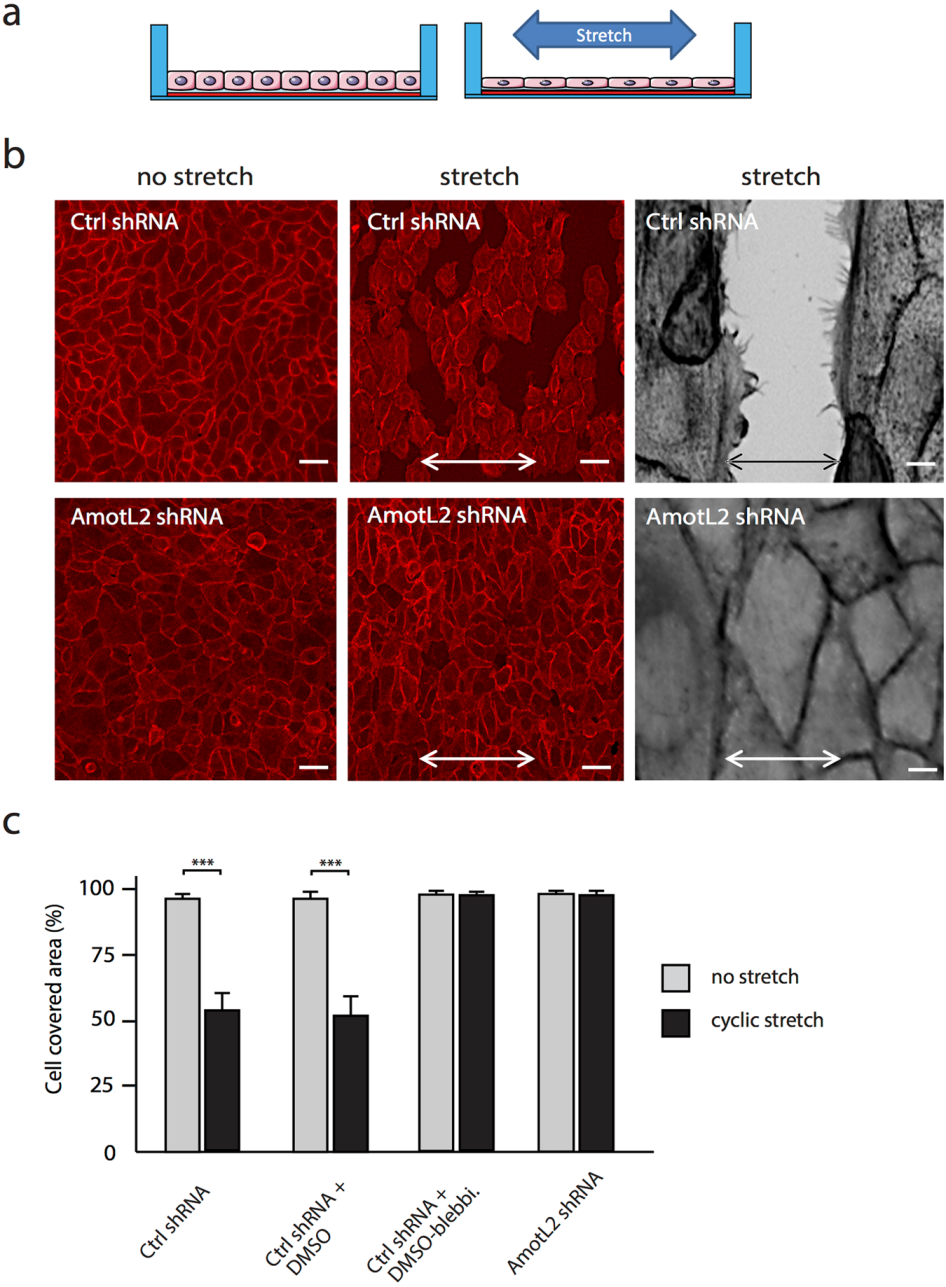
AmotL2 controls epithelial cell sheet elasticity. (**a**) Schematic figure of the cellular stretch assay. Cells were grown to confluency on fibronectin coated silicone membranes. The membranes were attached to a stretch apparatus and exposed to cyclic stretching for 2 hours with 30% amplitude and 0.25 Hz (stretching 15 times/min). (**b**) Ctrl or AmotL2 shRNA HaCaT cells under non-stretch or cyclic stretch conditions as indicated. Cells were visualized with phalloidin staining. Scale bars = 100 µm (grey) and 10 µm (red). (**c**) Quantification of cell coverage after exposure to cyclic stretch. Inhibition of actomyosin contractility using blebbistatin mimiced the effect of Amotl2 depletion, ***p < 0.001, The experiment was repeated six times.

Our findings indicated a role of amotL2 in the coupling of actin fibers to membrane anchors and thereby potentially connecting endogenous cellular tensile forces to neighboring cells. This also opened up the question whether this protein complex could integrate external mechanical forces and transmit them at a supra-cellular level. In order to address this issue, we used a cellular stretch assay (Fig. 5a). In this system HaCaT (human keratinocytes) cells were grown to confluency on fibronectin-coated elastic silicone membranes. Mechanical force was loaded on the membrane using a uniaxial cyclic stretch apparatus. After 2 hours of 30% cyclic stretch, approximately 50% of the control cells exhibited disruptions at the cellular junctions resulting in a discontinuous epithelial sheet (Fig. 5b and c). The actin filaments in the remaining cells reorganized perpendicular to the applied force. In contrast, the epithelial layer of the amotL2 shRNA cells remained intact (Fig. 5b and c). We used blebbistatin to investigate whether inhibition of actomyosin contractility could prevent the cell junction disruption in a similar fashion. A 15 min pre-treatment with blebbistatin completely prevented the mechanical stress induced junctional rupture in the stretch assay. These data indicated that amotL2 influences cell sheet topology by promoting actin-mediated cellular stiffness, allowing cellular adaptation to extracellular force. AmotL2 shRNA or blebbistatin treatment promotes a more fluid epithelium that is more resilient to mechanical stress due to higher elasticity.

### AmotL2 controls epithelial geometry in Zebrafish skin epithelium

The complex development of epithelial structures can be studied in zebrafish, which allows for an easy visualization of these processes at a cellular level *in vivo*. In zebrafish, *amotl2* is duplicated with paralogues on chromosomes 6 (*amotl2a*) and 2 (*amotl2b*)^28^.

**Figure 6:**
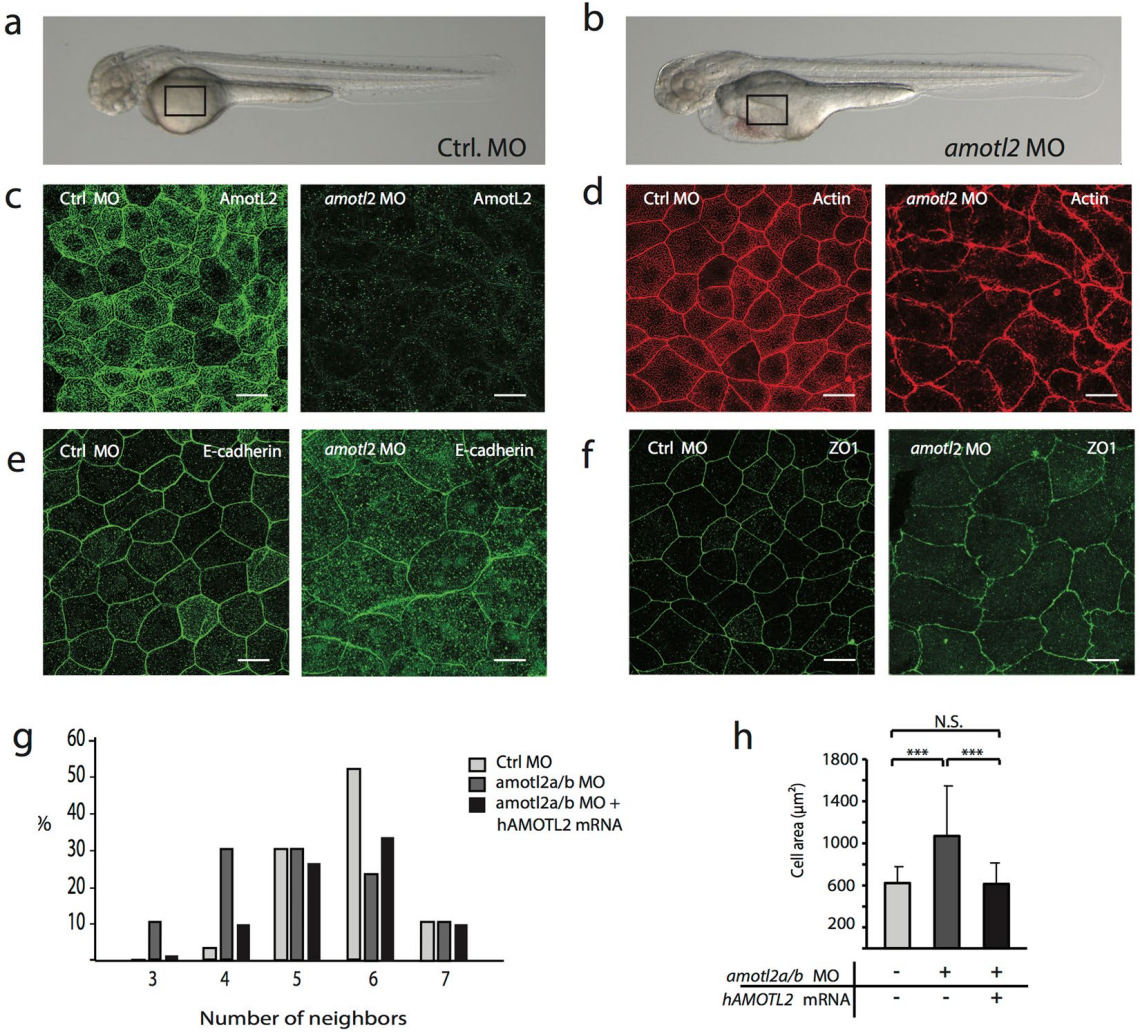
AmotL2 controls actin organization and epithelial cell geometry in zebrafish skin. (**a**) Bright-field image of a zebrafish embryo 48 hours post fertilization (hpf). (**b**) Bright-field image of a zebrafish *amotl2* morphant. The zebrafish development is apparently normal except for the pericardial edema due to vascular defects as described^29^. The black square indicates the area of the skin that is visualized in (**c**–**f**). Scale bars = 25 µm. (**c**–**f**). Confocal microscopy images of whole mount immunostainings of zebrafish skin. (**c**) Shows the localization of amotL2 in the control fish as indicated and knock-down efficiency after injection of *amotl2* MO. (**d**) Visualization of actin filaments using phalloidin staining. (**e**) and (**f**) E-cadherin as adhesion junction marker and ZO1 as tight junction marker. Scale bars = 25 µm, Data are derived from three independent experiments. (**g**) Quantification of number of neighboring skin cells in Ctrl MO, *amotl2* MO and *amotl2* MO + p100 mRNA (rescue experiment) zebrafish embryo skin. The control cells are mostly pentagonal and hexagonal, while cells with three or four corners were observed to a higher extent in the *amotl2* MO embryos. The phenotype could be rescued by co-injection of a human *AMOTL2* p100 mRNA. Differences in distributions between the three populations were evaluated by two-sample Kolmogorov-Smirnov test. Ctrl vs. *amotl2* MO, p-value = 2.07 × 10^−5^; *amotl2* MO vs. *amotl2* MO + p100 mRNA, p-value = 0.00029; ctrl vs. *amotl2* MO + p100 mRNA p-value = 0.024. N(Ctrl) = 241 cells, n(*amotl2* MO) = 228 cells, n(*amotl2* MO + p100 mRNA) = 373 cells. Data are derived from three independent experiments. (**h**) Bar diagrams summarizing the differences in average cell area size of Ctrl MO, *amotl2* MO and *amotl2* MO + p100 mRNA (rescue experiment) zebrafish embryo skin. Ctrl vs. *amotl2* MO, p-value = 4.35 × 10^−27^; *amotl2* MO vs. *amotl2* MO + p100 mRNA, p-value = 1.75 × 10^−34^; ctrl vs. *amotl2* MO + p100 mRNA, p-value = 0.64. N(ctrl) = 144 cells, n(*amotl2* MO) = 216 cells, n (*amotl2* MO + p100 mRNA) = 227 cells. Data are derived out of three independent experiments.

We used an anti-sense morpholino (MO) approach to target the translation initiation sites of both amotl2 paralogues. Bright-field images of control and *amotl2* MO*-*treated embryos at 48 hours post-fertilization are shown in Fig. 6a and b. The *amotl2* morphants suffered from pericardial edema due to circulatory defects as previously described (Fig. 6b)^28^. We could show amotl2 expression in zebrafish skin cells with whole mount immunofluorescent stainings by using the previously established amotL2 antibody (Fig. 6c)^28^. AmotL2 was confined to cellular junctions and cytoplasmic filamentous structures. The knock-down efficiency of the MO in skin cells was assessed by whole mount immunofluorescent staining and by qPCR as previously published (Fig. 6c)^28^. The effect on the cytoskeletal organization was analyzed by phalloidin staining. Similar to the epithelial amotL2 shRNA cells, junctional actin was clearly detectable whereas the non-junctional actin filaments were almost completely removed (Fig. 6d). E-cadherin staining was strictly localized to cell-cell junctions in control MO skin cells but more diffusely spread in the membrane and cytosol of skin cells in the *amotL2* morphants (Fig. 6e), as also earlier detected in the amotL2 shRNA epithelial cells (Fig. 1f). Junctional stainings using antibodies against ZO1 showed that the cellular surface area was almost doubled (Fig. 6f and h). Analysis of the skin epithelium also revealed a decrease in cells with hexagonal shape and an increase of cells with fewer sides towards their neighboring cells (Fig. 6g). Co-injection of the *amotl2* MO with human p100 *AMOTL2* mRNA could rescue the actin architecture in the skin cells (Supplemental Fig. 4) and restore geometry and cellular size (Fig. 6g and h). We concluded that amotL2 is essential for the packing of skin epithelial cells *in vivo*.

### AmotL2 is expressed in the human and mouse trophectoderm

**Figure 7:**
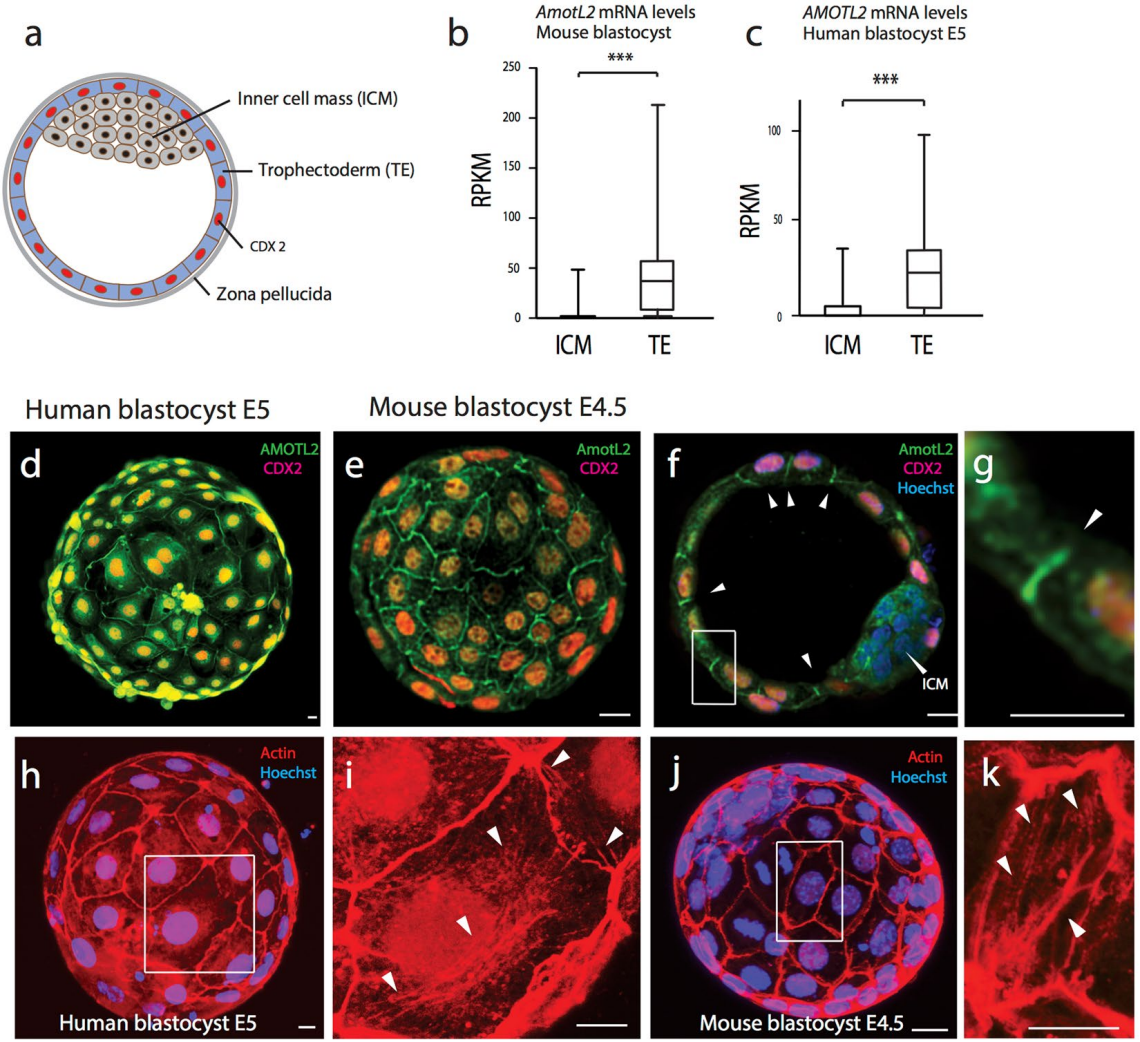
AmotL2 expression in mammalian blastocysts. (**a**) Schematic of mammalian blastocyst visualizing its characteristic components. The Inner Cell Mass (ICM) cells are located towards the inner side of the blastocyst. The outer TrophEctoderm (TE) cells express the TE specific transcription factor CDX2. The zona pellucida covers the entire blastocyst. (**b**) and (**c**) Bar diagrams summarizing *AMOTL2* mRNA expression level in inner cell mass (ICM) versus trophectoderm (TE) cells of mouse- and human- blastocysts. ***p < 0.001, 61 mouse and 215 human cells were analyzed from 9 embryos of each species. (**d**) and (**e**) Representative immunofluorescent staining of 6 human blastocysts (embryonic day 5) and 16 mouse blastocysts (embryonic day 4.5) to study localization of AmotL2. (**f**) An optical section of mouse blastocyst with Cdx2 negative ICM. AmotL2 signal is not detected in the ICM but restricted to the lateral side of TE cells (as indicated with white arrows). Total of 8 blastocysts were analyzed showing the same result. (**g**) Magnification of Figure **f**. showing AmotL2 localization to the lateral side of TE cells. (**h**–**k**) Immunofluorescent stainings of human blastocysts (embryonic day 5) and mouse blastocysts (embryonic day 4.5) to visualize actin fibers. The magnified blastocyst areas (as indicated) show radial actin fibers spanning throughout the cell and running perpendicular to the cellular junctions (as indicated with white arrows). 6 human and 11 mouse blastocysts were analyzed showing the same result.

The first specified cell-type to be established during mammalian development is the trophectoderm (TE) that belongs to the class of epithelium with tight and adherens junctions and exhibit cell polarization (Fig. 7a). The trophectoderm is specified through a positional mechanism where the outer cells sense that they are in an outer position. This positional sensing process has been shown to involve components of the Hippo signaling pathway together with amot and amotL2^29, 30^ to drive expression of the trophectoderm transcription factor Cdx2^43^. Ultimately, correct formation of the trophectoderm is crucial for normal embryo development as it mediates the implantation of the blastocyst into the uterus and gives later rise to the placenta^44^. Since amot together with amotL2 has been implicated in the segregation of the inner cell mass (ICM) from the trophectoderm^29, 30^ we reasoned that it would be of relevance to investigate the expression pattern of amotL2 and its potential role in regulating cell shape and lineage specification of the epithelial trophectoderm. Mining of recently published single-cell RNA sequencing data sets showed significantly higher expression of amotL2 in the trophectoderm of both human and mouse blastocysts compared to the inner cell mass (Fig. 7b and c)^45, 46^. Antibody staining confirmed this finding at a protein level and showed subcellular localization towards the lateral cell-cell junctions in the trophectoderm (Fig. 7d–g).

### AmotL2 is essential for radial actin fibers and cell shape of the blastocyst trophectoderm

Phalloidin stainings of human and mouse blastocysts confirmed the presence of both, junctional actin aligning along the cell junctions and radial actin fibers oriented perpendicular to the cellular junctions, spanning throughout the cytoplasm (Fig. 7h–k).

To investigate whether radial actin fiber maintenance in trophectoderm cells also depends on amotL2 expression, as observed in epithelial cell lines *in vitro* and in zebrafish *in vivo*, we silenced the amotL2 gene in mouse blastocysts. For this purpose we used a floxed mouse strain^28^ and injected *Cre* mRNA in the fertilized zygotes to excise the amotL2 gene. As a control, GFP mRNA was injected into the embryos. Injected embryos were culture *in vitro* and analyzed at early and late blastocyst stage. Successful excision of the *floxed* amotL2 was confirmed through antibody staining (Supplemental Fig. 5a).

**Figure 8:**
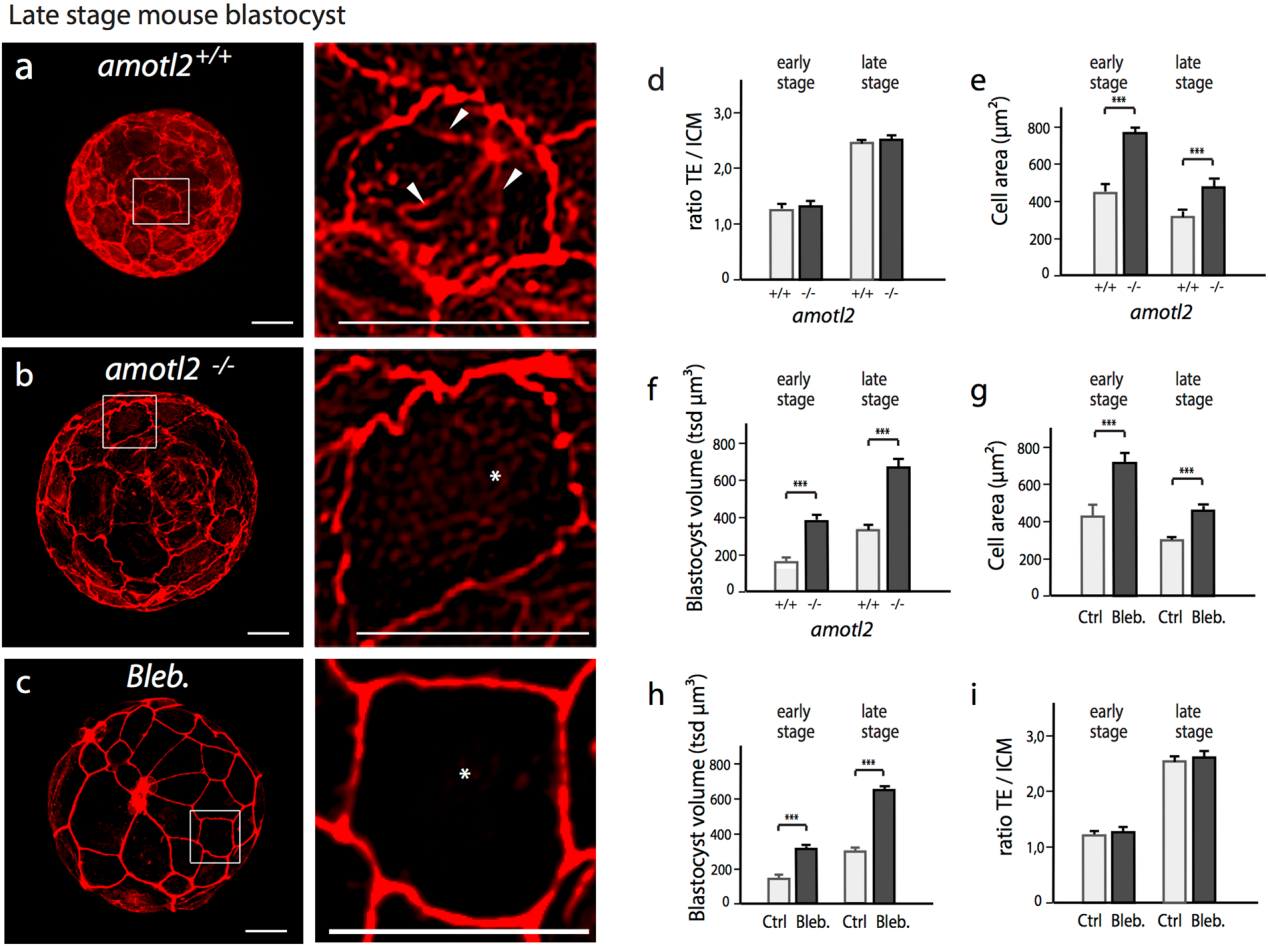
AmotL2 controls radial actin fiber formation in mouse blastocysts. (**a**–**c**) Actin immunofluorescent staining of *amotL2* ^+/+^, *amotL2* ^−/−^ and blebbistatin treated late stage mouse blastocysts. The magnified blastocyst areas (as indicated) show actin fibers in *amotL2* ^+/+^ and absence of actin fibers and enlarged cells in *AmotL2* ^−/−^ and blebbistatin treated blastocysts. Scale bars = 20 µm. (**d**–**i**) Bar diagrams quantifying TE-ICM ratio, cell area size, blastocyst volume of *amotL2* ^+/+^, *amotL2* ^−/−^ and blebbistatin treated mouse blastocysts at early and late stages. ***p < 0.001, 6 blastocysts were analyzed from each group.

As amot-family members have been implicated in trophectoderm versus inner cell mass specification^29, 30^ we first explored this issue. We stained control and *amotL2* ^−/−^ mouse blastocysts for the trophectoderm specific transcription factor CDX2, which was correctly expressed only in outer trophectoderm cells of both blastocyst cohorts (Supplemental Fig. 5b). We further analyzed the number of cells and the ratio of trophectoderm versus inner cell mass cells in *amotL2* ^+/+^ and *amotL2* ^−/−^ blastocysts, which was equal in both populations (Fig. 8d, Supplemental Fig. 5c). Turning focus towards the blastocyst actin cytoskeleton we could detect a distinct absence of radial actin fibers in *Cre* injected mice whereas the junctional actin remained intact (Fig. 8a and b). In addition to the loss of radial actin fibers, *Cre* mRNA injection resulted in approximately 60% increased cell area size of trophectoderm cells contributing to an increased blastocyst volume (Fig. 8b, e and f).

To further support that the observed changes in cell area size and volume were dependent on remodeling of radial actin fibers, we incubated mouse blastocysts with blebbistatin (100 μM blebbistatin for 6 hours). This treatment phenocopied *Cre* mRNA injected blastocysts resulting also in increased cell area size and volume without affecting cell numbers (Fig. 8c,g–i and Supplemental Fig. 5c).

**Figure 9:**
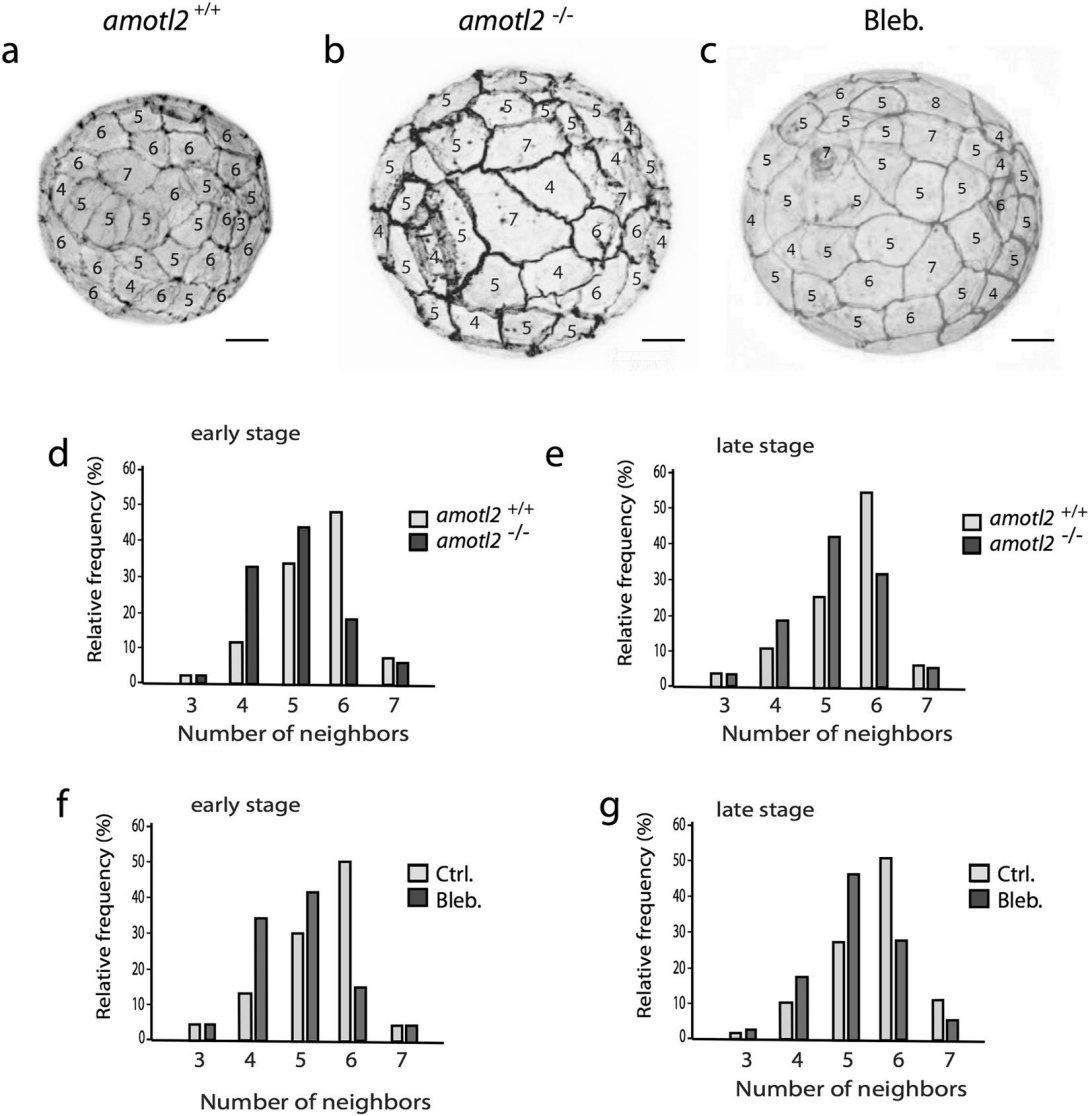
AmotL2 is essential for TE geometrical packing in mouse blastocysts. (**a**–**c**) Gray-scale actin immunofluorescent staining of *amotL2* ^+/+^, *amotL2* ^**−/−**^ and blebbistatin treated late stage blastocysts. Clearly visible are the cellular TE junctions with the number of cell sides indicated for each cell. scale bar = 20 µm. (**d**–**g**) Quantification of number of neighboring cells in *amotL2* ^+/+^, *amotL2* ^**−/−**^ or blebbistatin treated early and late stage mouse blastocysts. The differences in the number of neighbors were statistically analyzed using the two-sample Kolmogorov-Smirnov test. *AmotL2* ^+/+^ vs. *AmotL2* ^**−/−**^ early- and late stage blastocysts, p < 0.001; Ctrl KO vs. blebbistatin treated early- and late stage blastocysts, p < 0.001. 6 blastocysts were analyzed from each group.

To further investigate if the loss of amotL2 and actin fibers would also impact the hexagonal cell shape of trophectoderm cells, we evaluated 3D images of control and amotL2 excised blastocysts at early and late blastocyst stage. The most abundant cell shape of control blastocyst trophectoderm cells was hexagonal (Fig. 9a,d and e). In contrast, amotL2 excised blastocysts were mainly assembled out of trophectoderm cells comprising a pentagonal cell shape (Fig. 9b,d and e). Again, similar results were observed following pharmacological disruption of actin fibers using blebbistatin treatment (Fig. 9c,f and g).

### AmotL2 is required for blastocyst hatching

**Figure 10:**
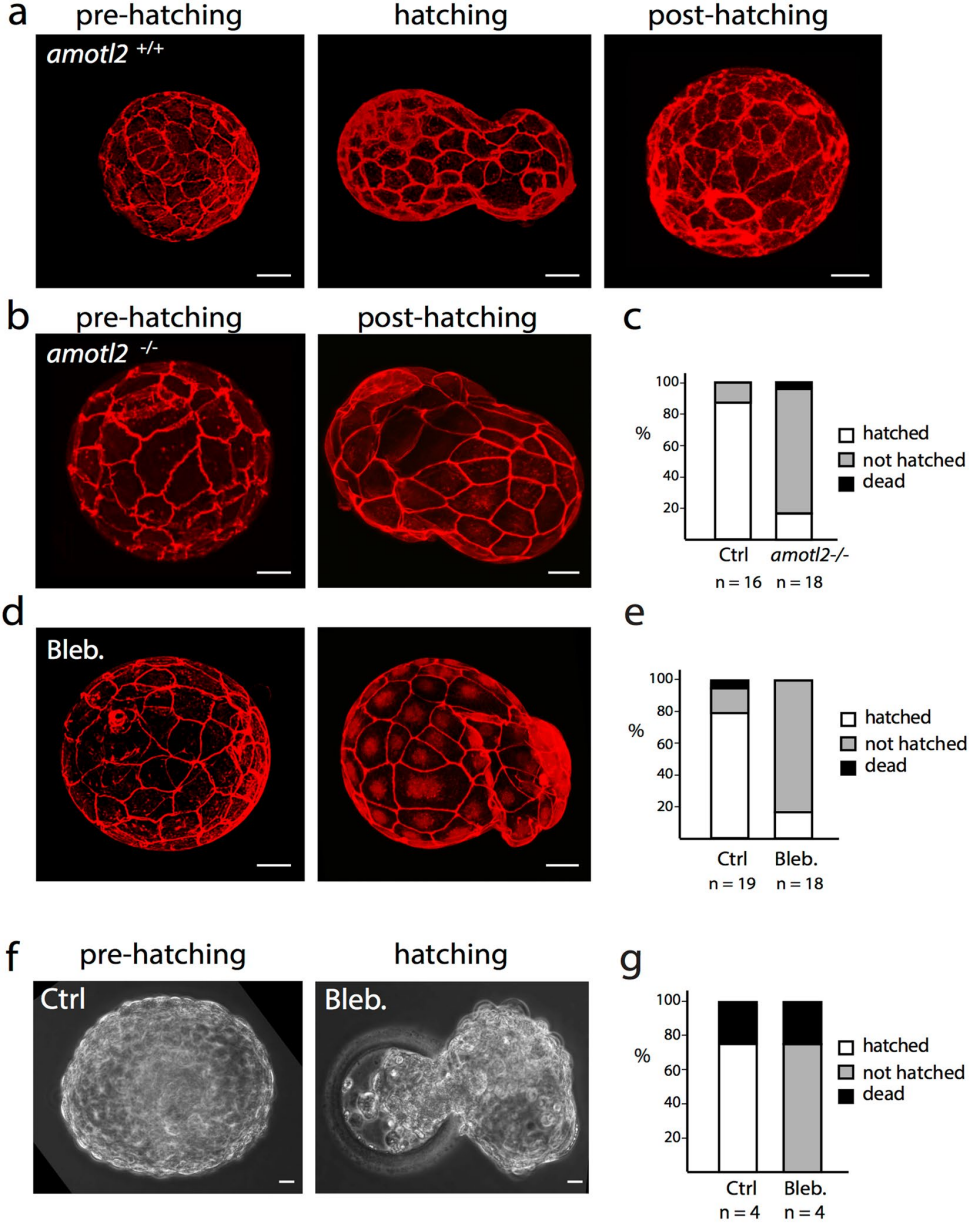
AmotL2 is required for blastocyst hatching. (**a**), (**b**) and (**d**) Actin immunofluorescent stainings of *amotL2* ^+/+^, *amotL2* ^**−/−**^ and blebbistatin treated late stage mouse blastocysts before, during or after the hatching process. Scale bars = 20 µm. (**c**–**e)** Bar diagrams summarizing the number of *amotL2* ^+/+^, *amotL2* ^**−/−**^ or blebbistatin treated blastocysts that hatched, failed to hatch or died during the experiment. Bar diagrams visualizing the pooled result of three independent experiments. The numbers of analyzed blastocysts (n) are stated under the bars. (**f**) Phase-contrast images of a Ctrl or blebbistatin treated late stage human blastocyst. Scale bars = 20 µm. (**g**) Bar diagrams summarizing the number of Ctrl KO or blebbistatin treated human blastocysts which hatched, failed to hatch or died during the experiment. The total amounts of analyzed blastocysts (n) are stated under the bars in the figures.

The mammalian embryo is enveloped by the zona pellucida, a glycoprotein extracellular shell, that prevents premature implantation into the uterine wall^47, 48^. When the embryo is ready to implant it must therefore first hatch through the zona pellucida before attaching to the endometrium. This process is mediated by combined enzymatic digestion of the matrix together with expansion and contraction cycles that drive the blastocyst out of the shell^49, 50^. As we have detected a loss of radial actin fibers in the mouse blastocyst after amotL2 excision, we explored whether these radial actin fibers are required for the process of hatching as described earlier^51, 52^. Indeed, during *in vitro* culture, 90% of all control blastocysts had hatched whereas only 20% of the amotL2 excised embryos completed the same process but were trapped in an intermediate hatching state (Fig. 10a,b and c).

Again, pharmacological disruption of actin filaments during the same period using blebbistatin reproduced the hatching phenotype (Fig. 10d and e). Finally, similar results were observed in blebbistatin treated human embryos where 75% of the control blastocysts hatched whereas 75% of the blebbistatin treated blastocysts remained in the hatching state (Fig. 10f and g) suggesting a conserved function also in humans.

## Discussion

The maintenance of epithelial cellular shapes depends on the ability of cells to relay and sense mechanical forces. Our report provides mechanistic insight on how these forces may be organized by the formation of cell-cell contacts. We show that amotL2 links E-cadherin to cytoskeletal actin and thereby affects the cytoskeletal organization, cell geometry and cell topology of epithelial cells *in vitro* and *in vivo*.

We provide evidence that amotL2 is essential for the development of specific actin fibers that connect in a perpendicular fashion to the outer membrane. These radial actin fibers are anchored in the membrane by coupling to the E-cadherin/AmotL2/MAGI1 ternary protein complex. Therefore, the amotL2 complex differs from the EPLIN/E-cadherin complex that connects junctional actin to the membrane^53, 54^. In contrast, reduction of amotL2 levels in epithelia *in vitro* and *in vivo* results in the loss of radial actin fibers. Our observations suggest that amotL2 organizes actin and thereby physical properties of epithelial sheets. This was supported by the *in vitro* stretch experiments, which pointed to a significant change in physical properties, most likely due to the loss of radial actomyosin filaments. The importance has previously been highlighted in model systems of monolayer epithelial cells without matrix attachment, where reducing actin depolymerized with Latrunculin B decreased cell sheet stiffness by approximately 50%^55^.

The organization of epithelial cells into hexagonal shapes is highly evolutionary conserved^35, 36^. This appears to be the optimum method of packaging cells in regards to transducing force and minimizing energy expenditures. The integration of cells in hexagonal patterns is also of functional importance. Hexagonal packing in the vertebrate lens minimizes light scattering by plasma membranes, controls the orientation of cell division as well as planar cell polarity and the organization of cilia. Previous studies have indicated the importance of cadherin-mediated cell-cell contacts and actomyosin contractility in controlling cell geometry^56–58^. Recent work has also provided evidence that tensile stress in the cellular cortex control hexagonal packing. Relaxation of the contractile force in the junctional actin resulted in lengthening of the adherens junctions and altered cell geometry^59^. Our observations suggest that amotL2 expression is critical for maintaining cellular geometry by supporting contractile forces required for the shaping of cuboidal epithelial cells and secondly by controlling hexagonal packing of cells in planar epithelium. Although it is not entirely clear how amotL2 affects the epithelial geometry, it is conceivable that either the loss of contractile actin filaments running perpendicular to the membrane is the cause of the loss of geometry or that contraction of junctional actin is also affected. The latter is supported by the observed lengthening of junction in amotL2 depleted cells.

The function of amot family members has previously been explored in the mouse blastocyst linking amot and amotL2 to Hippo signaling-dependent segregation of trophectoderm and inner cell mass^29, 30^. In these studies the inner cell mass cells of amot and amotL2 double-targeted embryos failed to fully mature the inner cell mass cells with ectopic expression of the trophectoderm marker gene *Cdx2*. This phenotype was not evident in our study where only amotL2 was targeted indicating overlapping and redundant function with amot in controlling trophectoderm versus inner cell mass maturation.

Since the change in cell shape has not been described in amot null embryos, our data suggest a specific function of amotL2 in linking E-cadherin to radial actin fibers. This observation was further supported by the distinct subcellular localization of the two family members, where amot is localized to the apical domain of trophectoderm cells^30, 51^ whereas we detect amotL2 expression at the lateral side of trophectoderm cells.

It is becoming increasingly clear that the mechanisms that control mouse and human preimplantation development is very poorly conserved, which can explain why mouse studies have not been all that helpful to improve human infertility treatments^60^. As the function of amotL2 appears to be universal between different types of epithelial cells from zebrafish to humans it is encouraging to see similar expression patterns of amotL2 as well as hatching phenotype in mouse and human embryos. This is further stressed by our detected shared hatching phenotype were both mouse- and human- blastocysts are trapped in an intermediate hatching state. This result suggests that the hatching process is controlled by orchestrate protease thinning of the zona pellucida together with proper biophysical characteristics of epithelial trophectoderm cells which we now show is dependent on amotL2 controlled radial actin fibers. We anticipate that amotL2 controlled epithelial properties will be important for many other epithelial structures throughout development and normal versus pathological physiology.

## Material and Methods

### Antibodies, probes

The following primary antibodies were used: actin (abcam, ab 3280); for human and dog cells: AmotL2 (polyclonal antibodies reactive to human AmotL2 C-terminal peptide, NH2- CLDSVATSRVQDLSDMVEILI -COOH); for zebrafish: AmotL2 (polyclonal antibody reactive to zebrafish amotL2 C-terminal peptide NH2- CQKAPSAVDLFKGVDDVSAE- COOH), custom made by Innovagen (Lund, Sweden); for mouse blastocysts: AmotL2 (GeneTex, GTX 120712; for human blastocysts: AmotL2 (Aviva, OAAB05455); α-catenin (BD, 610193); β-catenin (BD, 610154); CDX2 (BioSite, MU392A-UC); for human and dog cells: E-cadherin (BD, 610181); for mouse blastocysts: E-cadherin (Sigma, U3254); Ezrin (BD, 610602); GFP (Life technologies, A10262); MAGI1 (Sigma, WH0009223M3); Tubulin (Sigma, T5168); YAP1 (Santa Cruz, sc-101199); ZO-1 (Invitrogene, 339100). The following secondary antibodies were used: alexa fluor 405 anti-mouse (GE, A12380); alexa fluor 488 anti-mouse (GE, A11001); alexa fluor 488 anti-rabbit (GE, A11008); alexa fluor 488 anti-chicken (GE, A11039); alexa fluor 488 anti-rat (GE, A21208); alexa fluor 647 anti-mouse (GE, A31571); ECL anti-mouse IgG horseradish peroxidase (GE, NA931V); ECL anti-rabbit IgG horseradish peroxidase (GE, NA934V). To visualize the cellular actin fibers the F-actin probe Texas Red-X Phalloidin (Life technologies, T7471) was used. Nuclei were visualized using DAPI (Sigma, F6057) in epithelial cells and zebrafish skin and with Hoechst 33342 (Life technologies, H3570) in mouse- and human-blastocysts.

### Cell culturing and blebbistatin treatment

MDCK, Caco2 and HeLa cells were cultured in DMEM medium (Sigma, D6429) supplemented with 10% FBS and Penicillin/Strepotomycin.

To evaluate changes in cell area size, junction length or cell sides, cells were treated with 10 μM blebbistatin (Sigma, B0560) for 2 hours before experiment termination.

### Immunoflurescence, polarized epithelial cells

Polarized cells were grown on chamber slides (see also under “*polarization assay*”). To achieve 50% and 100% confluency 40 000 and 80 000 cells/cm^2^ was seeded overnight in chamber slides. Slides were washed for 5 s in phosphate buffered saline (PBS) and fixed in 4% para-formaldehyde (PFA) PBS for 10 min. Cells were washed for 3 × 5 min in PBS and permeabilized in 0.1% Triton-X100 PBS for 1 min. Slides were washed for 3 × 5 min in PBS and blocked for 1 hour in 5% horse serum (Gibco, 16050) PBS. Cells were incubated for 1 hour with the primary antibody in 5% horse serum PBS. Slides were washed 3 × 5 min in PBS and then incubated with the secondary immunofluorescent antibody (1:1000) and phalloidin (1:300) in 5% horse serum PBS. Slides were washed 3 × 5 min in PBS and mounted for microscopy with Fluoroshield including DAPI (Sigma, F6057). Images were captured with a Leica TCS SP5 confocal microscope.

### Lentiviral production and cell infection

Packaging vector pMD2.G, envelope vector pSPAX2 and viral cloning vector pLKO.1-TRC as pLKO.1 scramble shRNA vector were purchased from Sigma-Aldrich. AmotL2 shRNA oligonucleotides targeting canine and human *AMOTL2* mRNAs were cloned into the pLKO.1-TRC vector. The target sequence for the dog amotL2 knockdown construct (MDCK cells) was 5′-GCGGGAGAAAGAGGAGCAAATC-3′ and the target sequence for the human *AMOTL2* knockdown construct (CaCo2 and HaCaT cells) was 5′-GCGAGAGAAGGAGGAGCAGATC-3′. Lentiviral particles were produced by transfecting HEK293T cells with the pMD2.G, the pSPAX2 and the pLKO.1-TRC cloning vector containing either the amotL2 shRNA constructs or the scrambled shRNA constructs. Viral particles were collected after 48 hours of transfection, supplemented with 8 µg/ml protamine sulfate and used to infect target cells.

### Western blot

Cells were lysed in the following buffer (50 mM Tris-HCL pH 7.6, 150 mM NaCl, 1 mM EDTA, 1% Nonident P40, 1 x protease inhibitor (Roche, 04693159001) in dH_2_O). Lysates were prepared with SDS sample buffer (Novex, 1225644) containing 10% sample reducing agent (Novex, 1176192). Proteins were fractionated in a polyacrylamide Bis-Tris 4–12% gradient precast gel (Novex, NP0322BOX). Afterwards, proteins were transferred to a nitrocellulose membrane (Whatman, 10401396). The membrane was blocked in 5% non-fat milk, 0.1% Tween 20 in PBS and incubated with the primary antibody either for 1 hour at R.T. or overnight at 4 °C. The membrane was thereafter incubated for 1 hour at R.T. with an adequate horseradish peroxidase-conjugated secondary antibody. Labeled proteins were detected with chemiluminescence (ECL; Amersham, RPN2232).

### Immunoflourescence, zebrafish embryos

Zebrafish embryos were fixed in 4% PFA at 4 °C overnight. Embryos were washed 4 × 5 min in 0.1% Tween20 PBS and permeabilized in 0.5% TritonX-100 PBS for 30 minutes. Blocking was performed for 2 h in blocking buffer (5% Normal Goat Serum (NGS), 0.1% TritonX-100, 1% Bovine Serum Albumin (BSA), 0.1% Tween20 in PBS). Embryos were incubated with the primary antibody diluted in blocking buffer at 4 °C overnight. Next day embryos were washed 6 × 20 min in 0.1% Tween20 PBS and further incubated with the secondary antibody as described for the primary. Finally, washing was performed 6 × 40 min in 0.1% Tween20 PBS. To be able to mount the embryos the yolk sac was opened dorsally with a syringe to release the yolk. Immunofluorescence images were taken with a confocal microscope of the outer layer of yolk sac skin cells.

### Immunoprecipitation analysis

MDCK WT cells were used and transiently transfected with the constructs stated in the figures. 24 hours after transfection cells were lysed in the lysis buffer (for immunoprecipitation in Fig. 5A and D) (150 mM NaCl, 50 mM Tris-HCL pH 7.6, 10% Glycerol, 1,5 mM MgCl_2_, 1 mM EDTA, 1% Triton X-100, 1 x protease inhibitor (Roche, 04693159001) (for immunoprecipitation in Fig. 5 C) (150 mM NaCl, 50 mM Tris-HCL pH 7.6, 1% Nonident P40, 1 x protease inhibitor (Roche, 04693159001). Lysates were incubated with an antibody against AmotL2 or for control with rabbit immunoglobulins for 2 hours under rotation at 4 °C. Protein G Sepharose beads (GE, 17–0618–01) were added for additional 2 hours. Beads were washed 4 times with lyses buffer and heated for 10 min at 95 °C in 2 x LDS sample buffer (Novex, 1225644) containing 10% sample reducing agent (Novex, 1176192). The samples were fractionated by SDS PAGE and subsequently western blotted. Fractions of whole cell lysates were western blotted for evaluation of IP protein input level.

### Mechanical stretch assay

2 × 2 cm^2^ inner dimensions silicon chambers (LEBO production AB, HT6240025) with newly attached bottoms (self-made) were sterilized and 300 000 cells (AmotL2 shRNA knockdown HaCaT cells or scrambled shRNA knockdown HaCaT cells) were seeded in each chamber in DMEM containing 10% FBS, Penicillin/Strepotomycin and Fungizone. Cells were grown for 12 hours to confluent cell sheets. Some Ctrl KD and AmotL2 KD cells were treated with 100 μM blebbistatin or blebbistatin vehicle control DMSO (Sigma-Aldrich, D2650) for 15 minutes before the start of cyclic stretching. Cell coated silicon chambers were attached to a stretch apparatus exerting uniaxial cyclic stretch as previously described (K. Zeller *et al*., 2013, PLOS One 8, e64897). Cells were exposed to cyclic stretching for 2 hours with 30% amplitude and 0.25 Hz (stretching 15 times/min) in the cell culture incubator and with a stretch direction from left to right.

### *AMOTL2* mRNA level in blastocysts

Human and mouse single-cell RNA sequencing data from was mined for expression of *AMOTL2*. Individual cells were isolated from embryonic day (E5.0) human embryos and 64 cell mouse blastocysts. ICM/TE cells were extracted out of 9 mouse or human blastocysts. Expression of *AMOTL2* in the inner cell mass (human blastocyst, n = 73; mouse blastocyst, n = 33) and trophectoderm (human blastocyst, n = 142; mouse blastocyst, n = 28) was assessed using an Unpaired two-tailed t-test (GraphPad Prism version 6.0 d for Mac OS X, GraphPad Software, La Jolla California USA, www.graphpad.com).

### Zebrafish morpholino anti-sense experiments

Zebrafish were housed and mated in the Karolinska Institute zebrafish facility under standard conditions and in agreement with the regulations set out by the Swedish Board of Agriculture for the use of laboratory animals in scientific research. All experiments were performed in accordance with the relevant guidelines and regulations. All experimental protocols were approved by the regional ethics board (Jordbruksverket.se). For the knock-down *amotL2* in zebrafish embryos the following morpholino oligonucleotides were used:

Morpholino knockdown of *amotL2b*: 5′ TGAGTATTTATGATCTGAGCTGAAC 3′; Morpholino knockdown *amotL2a*: 5′ CTGATGATTCCTCTGCCGTTCTCAT 3′

Control morpholino *amotL2* mismatch: 5′ CCTCTTACCTCAGTTACAATTTATA 3′. Morpholinos were purchased from Gene Tools (Philomath, Oregon, USA). AmotL2b MO was injected at 3 ng/embryo and the *amotL2a* MO was injected at 1.5 ng/embryo.

For rescue experiments, a mRNA encoding human *AMOTL2* were synthesized using the SP6 Message Machine kit (Ambion, Austin, TX, USA), and 50 pg per embryo were co-injected together with the morpholinos. Morpholino-injected zebrafish embryos were maintained at 28 °C in standard E3 water supplemented with 0.003% phenyl-2-thiourea (PTU). Embryos were fixed in 4% PFA PBS at 28hpf for cell area analysis and at 34 hpf quantification of cell side distribution.

### AmotL2 null mouse zygotes production and experiments

Animal experiments were performed according to a permit from the Stockholm South ethical committee in Sweden (s-144-12 and s-258-15). As previously described *AmotL2* ^lox/lox^ mice were crossed with ROSA26-EYFP reporter mice^28^. *AmotL2* ^lox/lox^/ROSA26–EYFP females were superovulated with pregnant mare serum gonadotropin (PMSG) and on the next day with human chorionic gonadotropin (hCG) before mating. Harvested zygotes were injected with *Cre* or *GFP* mRNA. Cre-mRNA (pCS2 Cre-NLS)^61^ or EGFP mRNA (pCS2 EGFP)^58^ were transcribed using the Ambion Sp6 Message Machine kit (Fisher Scientific, AM1340) and diluted with injection buffer to a concentration of 50 ng/μl injection-buffer (10 mM Tris HCl pH 7,4; 0,2 mM EDTA). Injections were performed using Nikon TE200 microscope, with a differential interference contrast (Normarski) module and injector from Warner Instruments (PLI-100A). For experiments with wild type embryos, CD1 female mice (7–9 weeks) were crossed with CD1 male and embryos were flushed from the oviducts or uteri with M2 medium (Millipore, MR-015-D) and cultured in KSOM medium (Millipore, MR-121-D) in a 37 °C, 5% CO_2_ incubator. All methods were carried out in accordance with relevant guidelines and regulations. All experimental protocols were approved by the Swedish Board of Agriculture.

### Human embryos

were obtained from the Huddinge Karolinska Hospital/Sweden with informed consent from all the patients. The embryo studies and experimental protocols were approved by Karolinska Hospital, regional ethical review board EPN (2012/1765-31/1). The embryos were collected on embryonic day (E) 4.0 and cultured until E5.0 using standard conditions as performed in the IVF Clinic (5% CO_2_, 5% O_2_ in IVF plates containing 700 μl of CCM^TM^ media (Vitrolife) covered with 300 μl of Ovoil^TM^ (Vitrolife). All the methods were carried out in accordance with relevant guidelines and regulations.

### Immunoflourescence of blastocysts

Human or mouse blastocysts were fixed for 10 min in 4% PFA, washed by 3 × 10 min in blocking buffer (0.1% Tween20 and 4% FBS in PBS), permeabilized with 0.3% TritonX-100 PBS for 5 min and blocked o.n. at 4 °C in blocking buffer. Embryos were then stained with primary antibody o.n. at 4 °C, washed 3 × 10 min stained with a secondary antibody and/or phalloidin o.n. at 4 °C before final washes including nuclear staining with Hoechst 33342 (Life technologies, H3570) (1:500). Blastocysts were mounted using secure-seal spacer (ThermoFisher, S24737) between two microscope coverslips. Images were captured with a Zeiss LSM710-NLO confocal microscope.

### Blastocyst TE cell surface, blastocyst volume and TE/ICM ratio calculation

To evaluate the average TE cell surface of a blastocyst the average blastocyst diameter (_average_d) was calculated from the mean of the vertical and diagonal blastocyst diameter. In following the average TE cell surface area was determined by calculating the blastocyst surface with the formula A = π_ * average_d^2^ and then divide the value through the number of blastocyst TE cells.

To determine the blastocyst volume the formula V = ^1^/_6 *_ π _* average_d^3^ was used. To determine the TE/ICM ratio, all blastocyst TE cells were counted by counting all CDX2 stained nuclei (TE marker). All blastocyst cells were counted by counting all Hoechst stained nuclei. By subtracting the TE value from the all cells counted value, the ICM cell count was calculated.

### Rescue of the shAmotL2 phenotype

MDCK amotL2 shRNA knockdown cells and scrambled shRNA knockdown cells were plated in 8-well chamber slides in DMEM medium containing 1% Matrigel. After 30 min some of the MDCK amotL2 shRNA KD cell wells were transiently transfected with a human *AMOTL2* p100-expressing cDNA and a GFP-expressing cDNA to visualize cells which incorporated cDNA. 30 hours after transfection cells were fixed for 10 min with 4% PFA PBS and blocked for 1 hour in 10% horse serum (Gibco, 16050) PBS. Cells were stained with phalloidin according to the standard florescence staining protocol. Cell images were taken with a confocal microscope (Leica TCS SP5) to visualize the GFP signal and the actin staining. The cell area size of the AmotL2 p100-transfected amotL2 shRNA MDCK cells were evaluated by marking the GFP-labeled area with the “magic wand” tool in Adobe Photoshop (Adobe Photoshop CS5, version 12.0 × 32). By doing so, the pixel counts of the marked area were determined. By knowing the total pixel count of the entire image area as the size of the total image area the cell area of the GFP-expressing cell could be determent.

### Statistics

The T-Test was used for statistical evaluation if just two populations were compared. For statistical evaluation of cell shape distribution in Figs 3, 4 and 9 the Kolmogorov-Smirnov Test was applied. The following free statistical software was used: Wessa, P. (2013), Free Statistics Software, Office for Research Development and Education, version 1.1.23-r7, http://www.wessa.net/.

## Electronic supplementary material

The E-cadherin/AmotL2 complex organizes actin filaments required for epithelial hexagonal packing and blastocyst hatching

## References

[1] Pires-daSilva A, Sommer RJ (2003). The evolution of signalling pathways in animal development. Nat Rev Genet.

[2] Kim SH, Turnbull J, Guimond S (2011). Extracellular matrix and cell signalling: the dynamic cooperation of integrin, proteoglycan and growth factor receptor. J Endocrinol.

[3] Basson, M.A. Signaling in cell differentiation and morphogenesis. *Cold Spring Harb Perspect Biol* **4** (2012). 10.1101/cshperspect.a008151 PMC3367549 22570373

[4] Jaalouk DE, Lammerding J (2009). Mechanotransduction gone awry. Nat Rev Mol Cell Biol.

[5] Wozniak MA, Chen CS (2009). Mechanotransduction in development: a growing role for contractility. Nat Rev Mol Cell Biol.

[6] Schwartz MA (2010). Integrins and extracellular matrix in mechanotransduction. Cold Spring Harb Perspect Biol.

[7] Levental KR (2009). Matrix crosslinking forces tumor progression by enhancing integrin signaling. Cell.

[8] Mih JD, Marinkovic A, Liu F, Sharif AS, Tschumperlin DJ (2012). Matrix stiffness reverses the effect of actomyosin tension on cell proliferation. J Cell Sci.

[9] Gehler S (2009). Filamin A-beta1 integrin complex tunes epithelial cell response to matrix tension. Mol Biol Cell.

[10] Guo WH, Frey MT, Burnham NA, Wang YL (2006). Substrate rigidity regulates the formation and maintenance of tissues. Biophys J.

[11] Takeichi M (2014). Dynamic contacts: rearranging adherens junctions to drive epithelial remodelling. Nat Rev Mol Cell Biol.

[12] Rauzi M, Lenne PF, Lecuit T (2010). Planar polarized actomyosin contractile flows control epithelial junction remodelling. Nature.

[13] Levayer R, Lecuit T (2012). Biomechanical regulation of contractility: spatial control and dynamics. Trends Cell Biol.

[14] Levayer R, Pelissier-Monier A, Lecuit T (2011). Spatial regulation of Dia and Myosin-II by RhoGEF2 controls initiation of E-cadherin endocytosis during epithelial morphogenesis. Nat Cell Biol.

[15] Yao M (2014). Force-dependent conformational switch of alpha-catenin controls vinculin binding. Nat Commun.

[16] Buckley CD (2014). Cell adhesion. The minimal cadherin-catenin complex binds to actin filaments under force. Science.

[17] Ernkvist M (2008). Differential roles of p80- and p130-angiomotin in the switch between migration and stabilization of endothelial cells. Biochim Biophys Acta.

[18] Zheng Y (2009). Angiomotin-like protein 1 controls endothelial polarity and junction stability during sprouting angiogenesis. Circ Res.

[19] Wang Y (2011). Angiomotin-like2 gene (amotl2) is required for migration and proliferation of endothelial cells during angiogenesis. J Biol Chem.

[20] Zheng, Y. *et al*. Angiomotin like-1 is a novel component of the N-cadherin complex affecting endothelial/pericyte interaction in normal and tumor angiogenesis. **6**, 30622 (2016). 10.1038/srep30622 PMC4964570 27464479

[21] Wells CD (2006). A Rich1/Amot complex regulates the Cdc42 GTPase and apical-polarity proteins in epithelial cells. Cell.

[22] Yi C (2013). The p130 isoform of angiomotin is required for Yap-mediated hepatic epithelial cell proliferation and tumorigenesis. Sci Signal.

[23] Paramasivam M, Sarkeshik A, Yates JR, Fernandes MJ, McCollum D (2011). Angiomotin family proteins are novel activators of the LATS2 kinase tumor suppressor. Mol Biol Cell.

[24] Ranahan WP (2011). The adaptor protein AMOT promotes the proliferation of mammary epithelial cells via the prolonged activation of the extracellular signal-regulated kinases. Cancer Res.

[25] Ernkvist M (2006). p130-angiomotin associates to actin and controls endothelial cell shape. FEBS J.

[26] Gagne V (2009). Human angiomotin-like 1 associates with an angiomotin protein complex through its coiled-coil domain and induces the remodeling of the actin cytoskeleton. Cell Motil Cytoskeleton.

[27] Patrie KM (2005). Identification and characterization of a novel tight junction-associated family of proteins that interacts with a WW domain of MAGI-1. Biochim Biophys Acta.

[28] Hultin S (2014). AmotL2 links VE-cadherin to contractile actin fibres necessary for aortic lumen expansion. Nat Commun.

[29] Leung CY, Zernicka-Goetz M (2013). Angiomotin prevents pluripotent lineage differentiation in mouse embryos via Hippo pathway-dependent and -independent mechanisms. Nat Commun.

[30] Hirate Y (2013). Polarity-dependent distribution of angiomotin localizes Hippo signaling in preimplantation embryos. Curr Biol.

[31] Zhao B (2011). Angiomotin is a novel Hippo pathway component that inhibits YAP oncoprotein. Genes Dev.

[32] Wang W, Huang J, Chen J (2011). Angiomotin-like proteins associate with and negatively regulate YAP1. J Biol Chem.

[33] Ernkvist M (2009). The Amot/Patj/Syx signaling complex spatially controls RhoA GTPase activity in migrating endothelial cells. Blood.

[34] Mojallal M (2014). AmotL2 disrupts apical-basal cell polarity and promotes tumour invasion. Nat Commun.

[35] Gibson MC, Patel AB, Nagpal R, Perrimon N (2006). The emergence of geometric order in proliferating metazoan epithelia. Nature.

[36] Li Y, Naveed H, Kachalo S, Xu LX, Liang J (2012). Mechanisms of regulating cell topology in proliferating epithelia: impact of division plane, mechanical forces, and cell memory. PLoS One.

[37] Shutova M, Yang C, Vasiliev JM, Svitkina T (2012). Functions of nonmuscle myosin II in assembly of the cellular contractile system. PLoS One.

[38] Berx G, van Roy F (2009). Involvement of members of the cadherin superfamily in cancer. Cold Spring Harb Perspect Biol.

[39] Paredes J (2012). Epithelial E- and P-cadherins: role and clinical significance in cancer. Biochim Biophys Acta.

[40] Bratt A (2005). Angiomotin regulates endothelial cell-cell junctions and cell motility. J Biol Chem.

[41] Dobrosotskaya IY, James GL (2000). MAGI-1 interacts with beta-catenin and is associated with cell-cell adhesion structures. Biochem Biophys Res Commun.

[42] Sugihara-Mizuno Y (2007). Molecular characterization of angiomotin/JEAP family proteins: interaction with MUPP1/Patj and their endogenous properties. Genes Cells.

[43] Lanner F (2014). Lineage specification in the early mouse embryo. Exp Cell Res.

[44] Wiley LM, Kidder GM, Watson AJ (1990). Cell polarity and development of the first epithelium. Bioessays.

[45] Petropoulos S (2016). Single-Cell RNA-Seq Reveals Lineage and X Chromosome Dynamics in Human Preimplantation Embryos. Cell.

[46] Posfai, E. *et al*. Position- and Hippo signaling-dependent plasticity during lineage segregation in the early mouse embryo. *eLife* **6** (2017). 10.7554/eLife.22906 PMC5370188 28226240

[47] Vasioukhin V, Fuchs E (2001). Actin dynamics and cell-cell adhesion in epithelia. Curr Opin Cell Biol.

[48] Gupta SK (2012). Mammalian zona pellucida glycoproteins: structure and function during fertilization. Cell Tissue Res.

[49] Hammadeh ME, Fischer-Hammadeh C, Ali KR (2011). Assisted hatching in assisted reproduction: a state of the art. J Assist Reprod Genet.

[50] Cohen J, Feldberg D (1991). Effects of the size and number of zona pellucida openings on hatching and trophoblast outgrowth in the mouse embryo. Mol Reprod Dev.

[51] Cheon YP (1999). Role of actin filaments in the hatching process of mouse blastocyst. Zygote.

[52] Suzuki R, Niimura S (2010). Hatching and distribution of actin filaments in mouse blastocysts whose activities of protein kinase A were suppressed by H-89. J Reprod Dev.

[53] Taguchi K, Ishiuchi T, Takeichi M (2011). Mechanosensitive EPLIN-dependent remodeling of adherens junctions regulates epithelial reshaping. J Cell Biol.

[54] Abe K, Takeichi M (2008). EPLIN mediates linkage of the cadherin catenin complex to F-actin and stabilizes the circumferential actin belt. Proc Natl Acad Sci USA.

[55] Harris AR (2012). Characterizing the mechanics of cultured cell monolayers. Proc Natl Acad Sci USA.

[56] Izaguirre MF (2010). Role of E-cadherin in epithelial architecture maintenance. Cell Commun Adhes.

[57] Gemp IM, Carthew RW, Hilgenfeldt S (2011). Cadherin-dependent cell morphology in an epithelium: constructing a quantitative dynamical model. PLoS Comput Biol.

[58] Braga V (2000). Epithelial cell shape: cadherins and small GTPases. Exp Cell Res.

[59] Bardet PL (2013). PTEN controls junction lengthening and stability during cell rearrangement in epithelial tissue. Dev Cell.

[60] Niakan KK, Han J, Pedersen RA, Simon C, Pera RA (2012). Human pre-implantation embryo development. Development.

[61] Chazaud C, Yamanaka Y, Pawson T, Rossant J (2006). Early lineage segregation between epiblast and primitive endoderm in mouse blastocysts through the Grb2-MAPK pathway. Dev Cell.

