# Supplementary material for: The E-cadherin/AmotL2 complex organizes actin filaments required for epithelial hexagonal packing and blastocyst hatching
Source: Sci Rep. 2017 Aug 25;7:9540. doi: 10.1038/s41598-017-10102-w (PMC5572699; doi:10.1038/s41598-017-10102-w)

Supplementary information file for the manuscript:

The E-cadherin/AmotL2 complex organizes actin filaments required for epithelial hexagonal packing and blastocyst hatching.

Sebastian Hildebrand[1,2], Sara Hultin[2], Sophie Petropoulos[1], Aravindh Subramani[2], Yuanyuan Zhang[2], Xiaofang Cao[3], John Mpindi[4,5], Olli Kalloniemi[4,5], Staffan Johansson[3], Arindam Majumdar[2,6], Fredrik Lanner[1*#], and Lars Holmgren[2*#]

[1]Department of Clinical Sciences, Intervention and Technology (CLINTEC), Karolinska Institutet and Division of Obstetrics and Gynecology, Karolinska University Hospital, Huddinge; Sweden

[2]Department of Oncology-Pathology, Cancer Centrum Karolinska (CCK), Karolinska Institutet, Stockholm, Sweden

[3]Department of Medical Biochemistry and Microbiology,

Uppsala Biomedical Center (BMC), Uppsala University, Uppsala, Sweden

[4]Medical Biotechnology, VTT Technical Research Centre of Finland, Turku, Finland
[5]Institute for Molecular Medicine Finland (FIMM), University of Helsinki, Helsinki, Finland
[6]Present address: Eli Lilly and Company, Lilly Corporate Center, Indianapolis, IN 46285, USA

Running title: AmotL2 controls epithelial physiology

#Correspondence to Lars Holmgren, E-mail: lars.holmgren@ki.se and Fredrik Lanner, E-mail: fredrik.lanner@ki.se.

*Co-senior authors.

Supplementary Figure 1. Expression of amotL2 in epithelial cells and tissues. **a**. The GeneSapiens database was used to evaluate expression levels of amotL2 across normal and malignant human tissues. This database (http://www.genesapiens.org) covers the relative gene expression patterns for 19000 genes across all the 18899 annotated normal and pathological human tissue samples from publicly available Affymetrix microarray experiments. **b**. Relative mRNA expression levels of amotL2 in 755 cell lines from the Genesapiens database.

Supplementary Figure 2. Western blot analysis and phenotype of amotL2 shRNA depleted cells. Western blot analysis of amotL2 expression in Ctrl and amotL2 shRNA transfected Caco2 cells **a and** HaCaT cells **b**. **c.** The bar diagram shows cell area of MDCK cells at 50% confluency. Transfection of human amotL2 p100 expression construct rescues the cell shape in the canine MDCK cells. **d**. Actin filaments visualized using phalloidin staining of Caco2 (left panel) and HaCaT cells (right panel). Note the change in cell shape and loss of radial actin filaments.

Supplementary Figure 3. Depletion of amotL2 does not affect keratin or tubulin network organization. Ctrl or AmotL2 shRNA MDCK cells were stained with pan-keratin or α-tubulin antibodies as indicated. Scale bars=10 μm. Data are derived from three independent experiments.

Supplementary Figure 4. AmotL2 controls actin organization and epithelial cell geometry in zebrafish skin. Filamentous F-actin structures were lost in *amotl2* MO

epidermis, while junctional actin still seemed somewhat intact. Note the altered cell area and morphology in the skin of the *amotl2* MO embryo. The observed phenotype could be rescued by re-expression of human *AMOTL2* mRNA. Scale bars=10 µm. Data are derived from three independent experiments.

Supplementary Figure 5. Genetic deletion of amotL2 does not induce CDX2 expression in ICM cells or total cell number. a. The targeting strategy using cre-mRNA injection was confirmed using antibody staining for amotL2. In total 11 blastocysts were stained in each group. **b.** *AmotL2*$^{+/+}$ and *AmotL2*$^{-/-}$ late stage blastocysts were stained for the trophectoderm (TE) driving transcription factor CDX2 and with Hoechst 33342 dye to visualize cell nuclei. In *amotL2*$^{+/+}$ and *amotL2*$^{-/-}$ blastocysts CDX2 expression was only detectable in the outer cell monolayer but not in the ICM cells (dotted line). Scale bars=10 µm. 6 blastocysts were analysed in each group. **c.** No change in cell number was observed following amotL2 targeting or short-term blebbistatin treatment. 6 blastocysts were analysed in each group.

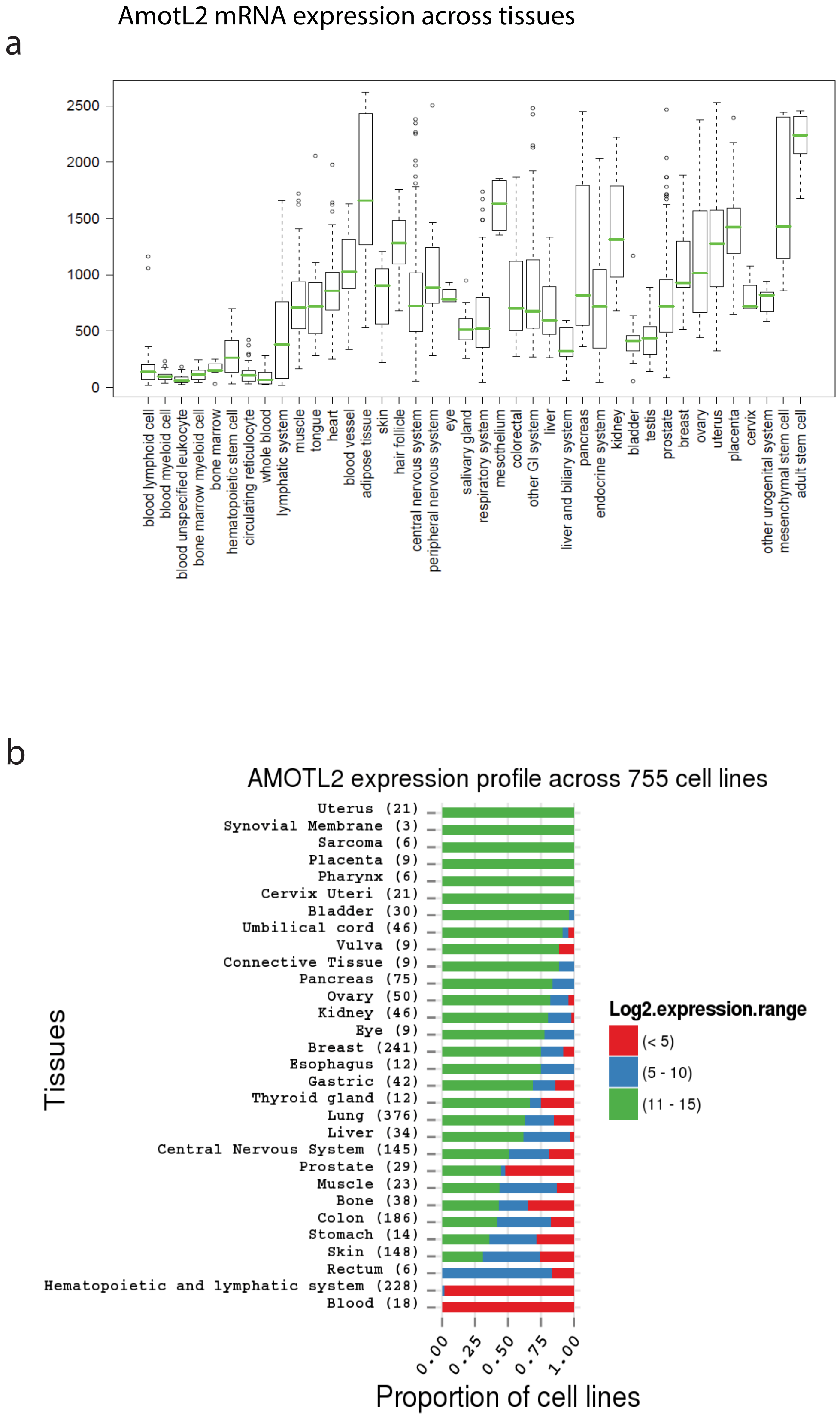

a
AmotL2 mRNA expression across tissues
2500
2000
1500
1000
500
0
blood lymphoid cell
blood myeloid cell
blood unspecified leukocyte
bone marrow myeloid cell
bone marrow
hematopoietic stem cell
circulating reticulocyte
whole blood
lymphatic system
muscle
tongue
heart
blood vessel
adipose tissue
skin
hair follicle
central nervous system
peripheral nervous system
eye
salivary gland
respiratory system
mesothelium
colorectal
other GI system
liver
liver and biliary system
pancreas
endocrine system
kidney
bladder
testis
prostate
breast
ovary
uterus
placenta
cervix
other urogenital system
mesenchymal stem cell
adult stem cell
b
AMOTL2 expression profile across 755 cell lines
Tissues
Uterus (21)
Synovial Membrane (3)
Sarcoma (6)
Placenta (9)
Pharynx (6)
Cervix Uteri (21)
Bladder (30)
Umbilical cord (46)
Vulva (9)
Connective Tissue (9)
Pancreas (75)
Ovary (50)
Kidney (46)
Eye (9)
Breast (241)
Esophagus (12)
Gastric (42)
Thyroid gland (12)
Lung (376)
Liver (34)
Central Nervous System (145)
Prostate (29)
Muscle (23)
Bone (38)
Colon (186)
Stomach (14)
Skin (148)
Rectum (6)
Hematopoietic and lymphatic system (228)
Blood (18)
0.00
0.25
0.50
0.75
1.00
Proportion of cell lines
Log2.expression.range
(< 5)
(5 - 10)
(11 - 15)

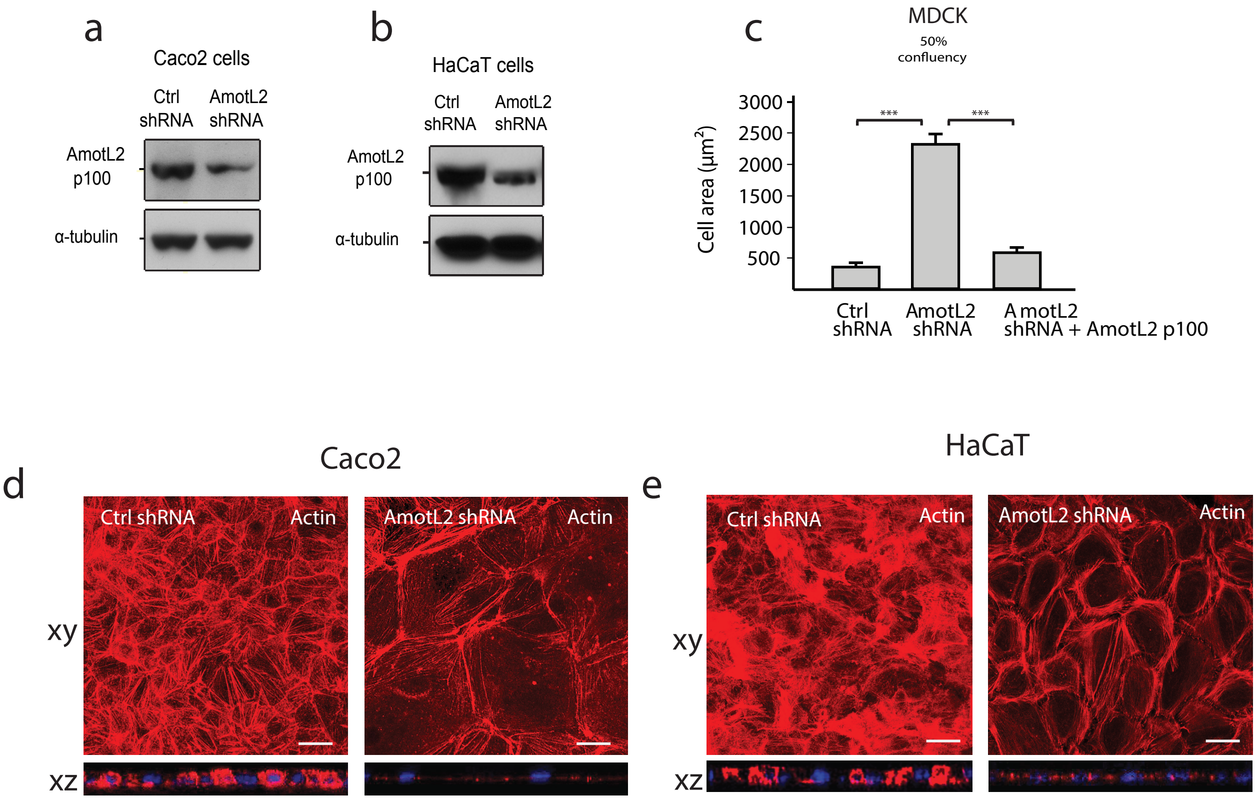
a
Caco2 cells
Ctrl shRNA
AmotL2 shRNA
AmotL2 p100
α-tubulin
b
HaCaT cells
Ctrl shRNA
AmotL2 shRNA
AmotL2 p100
α-tubulin
c
MDCK
50% confluency
Cell area (μm²)
3000
2500
2000
1500
1000
500
***
***
Ctrl shRNA
AmotL2 shRNA
AmotL2 shRNA + AmotL2 p100
d
Caco2
Ctrl shRNA
Actin
AmotL2 shRNA
Actin
xy
xz
e
HaCaT
Ctrl shRNA
Actin
AmotL2 shRNA
Actin
xy
xz

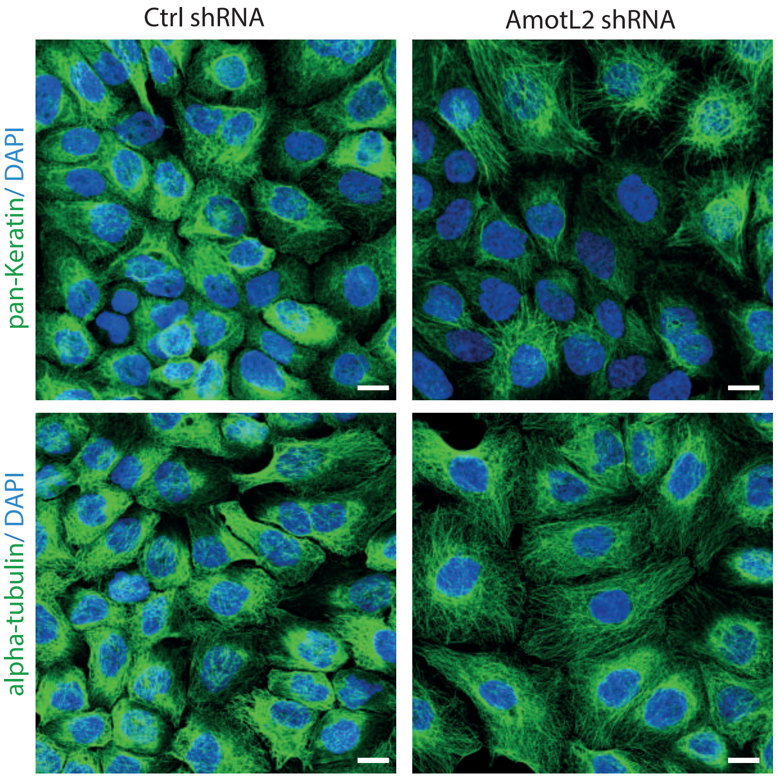
Ctrl shRNA
AmotL2 shRNA
pan-Keratin/ DAPI
alpha-tubulin/ DAPI

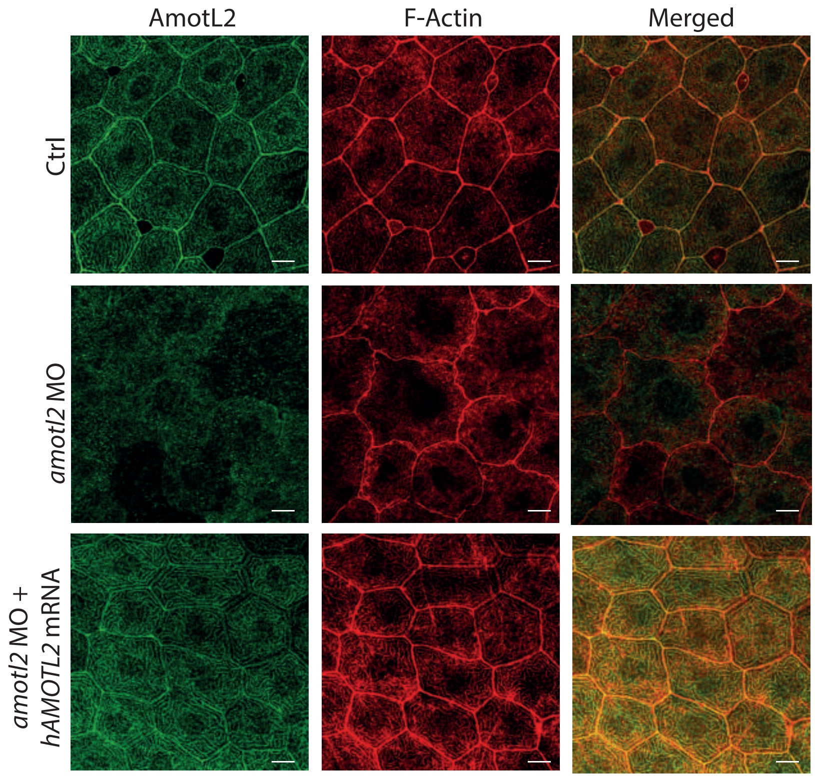
AmotL2
F-Actin
Merged
Ctrl
amotl2 MO
amotl2 MO +
hAMOTL2 mRNA

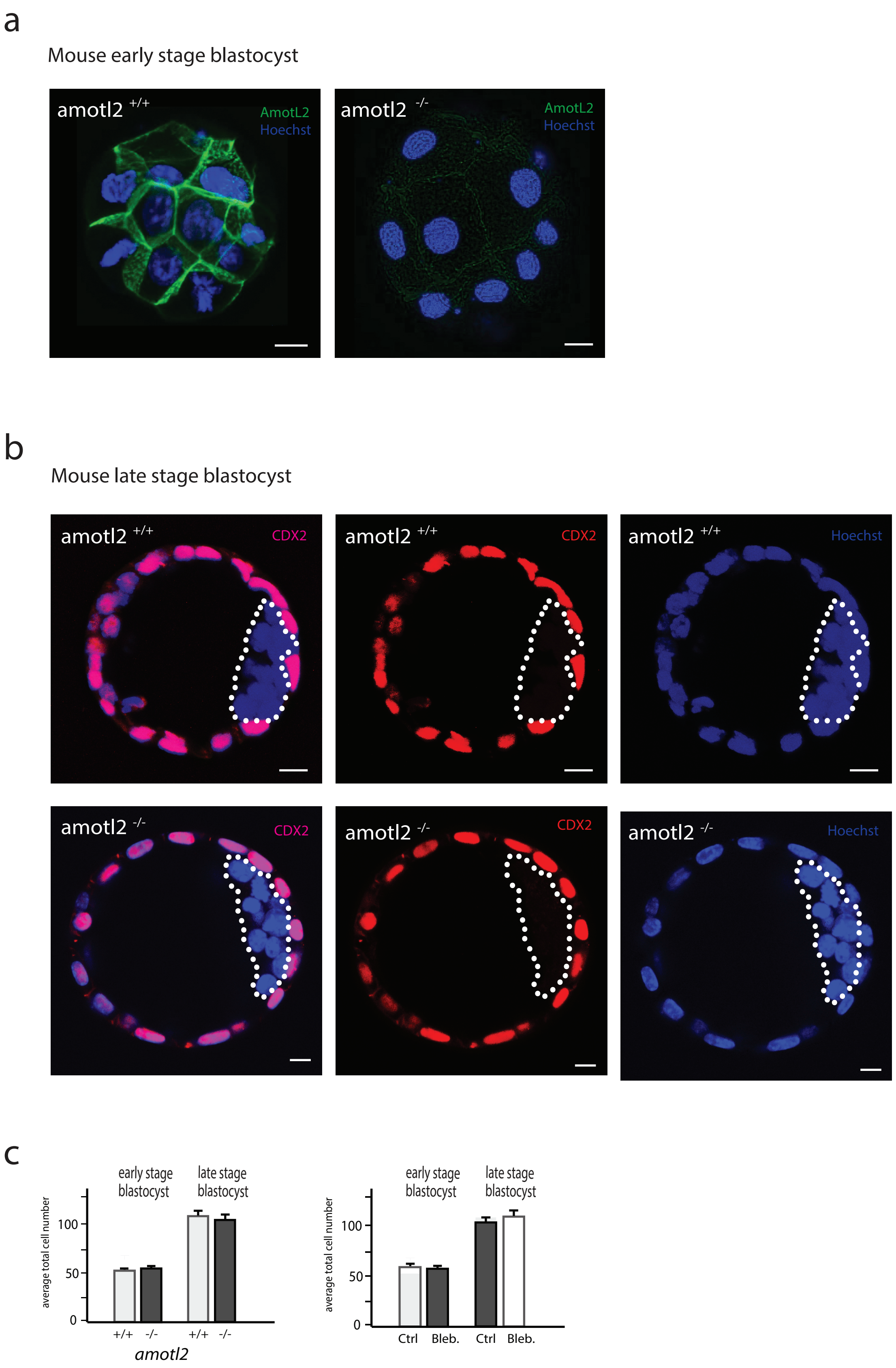
a
Mouse early stage blastocyst
amotl2 +/+
AmotL2
Hoechst
amotl2 -/-
AmotL2
Hoechst
b
Mouse late stage blastocyst
amotl2 +/+
CDX2
amotl2 +/+
CDX2
amotl2 +/+
Hoechst
amotl2 -/-
CDX2
amotl2 -/-
CDX2
amotl2 -/-
Hoechst
c
early stage blastocyst
late stage blastocyst
average total cell number
100
50
0
+/+
-/-
+/+
-/-
amotl2
early stage blastocyst
late stage blastocyst
average total cell number
100
50
0
Ctrl
Bleb.
Ctrl
Bleb.

Full blot to Figure 1b

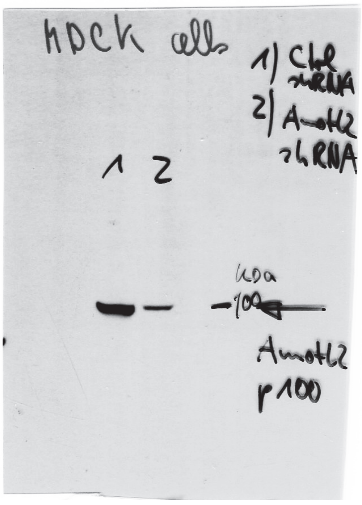

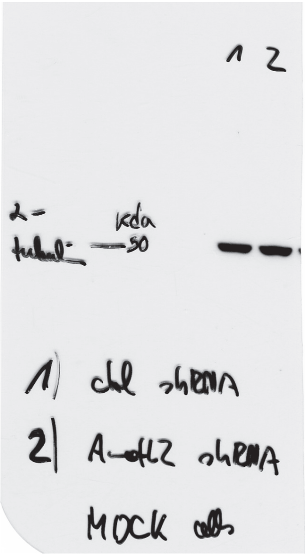

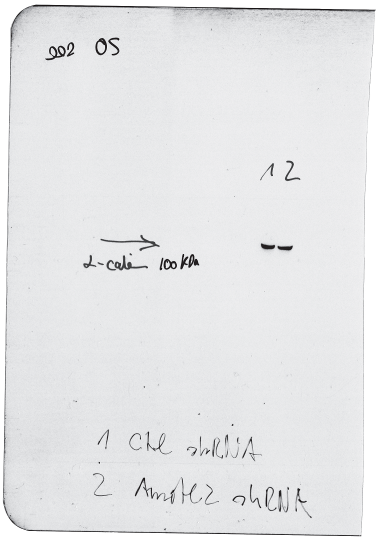

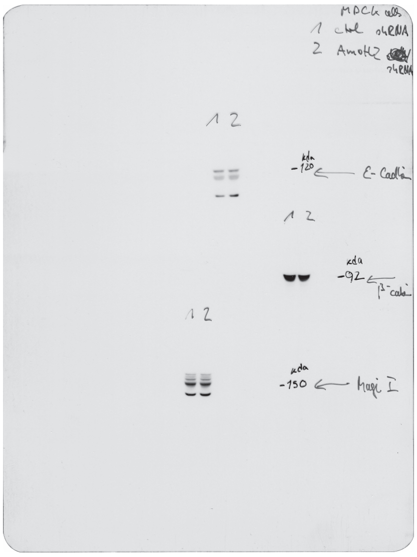

Full Blots to FIgure 4a

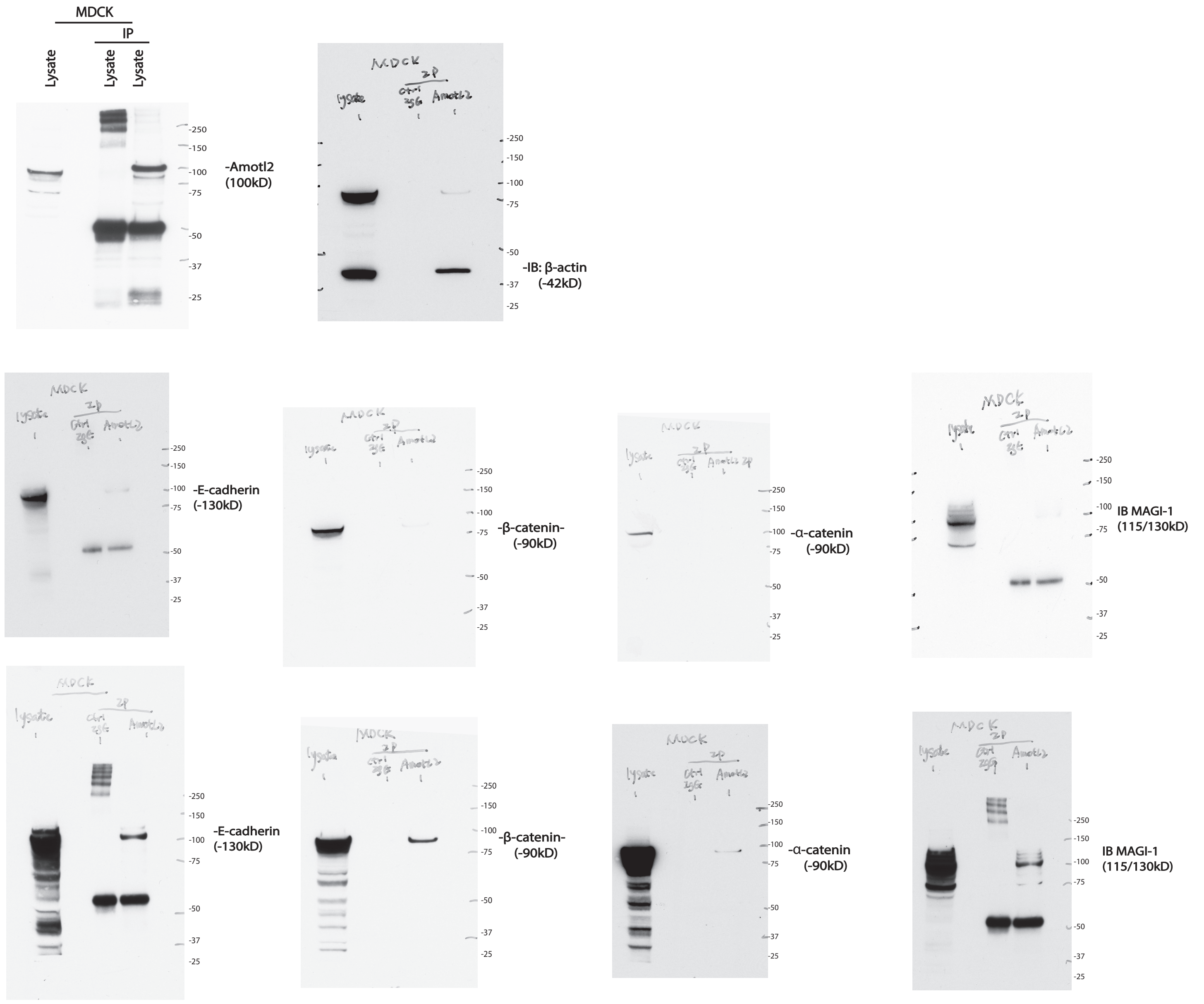

Full blot to Figure 4 C

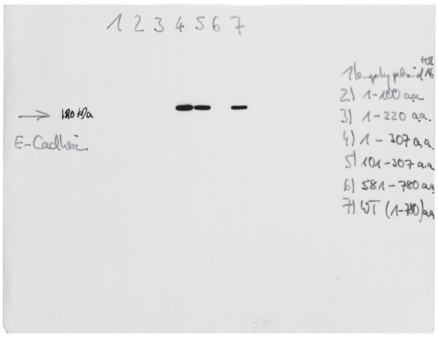

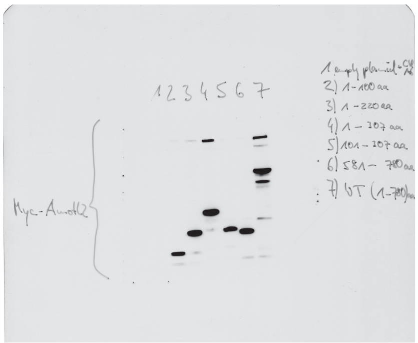

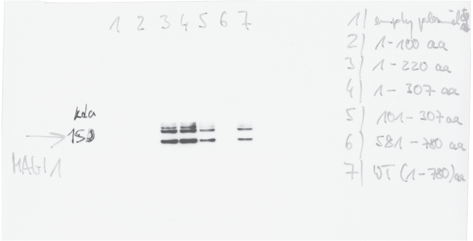

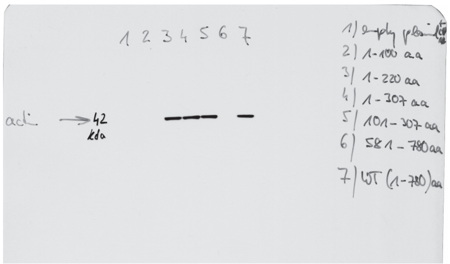

Full blot to Figure 4 D

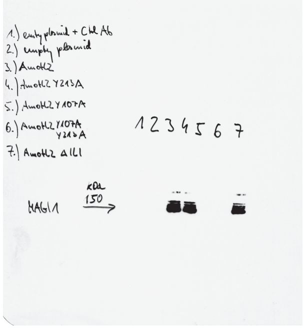

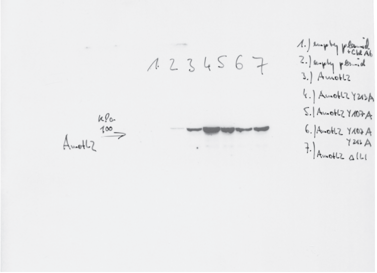

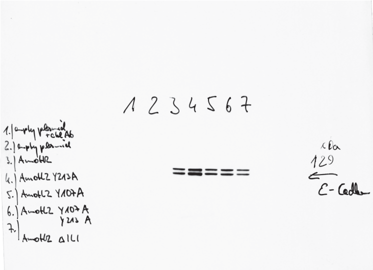

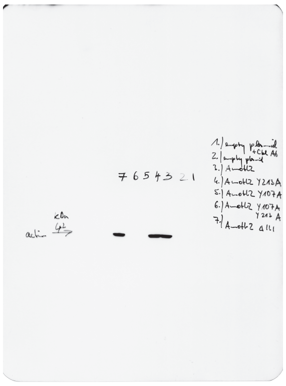

Full Full blot to supplementary Figure 2a

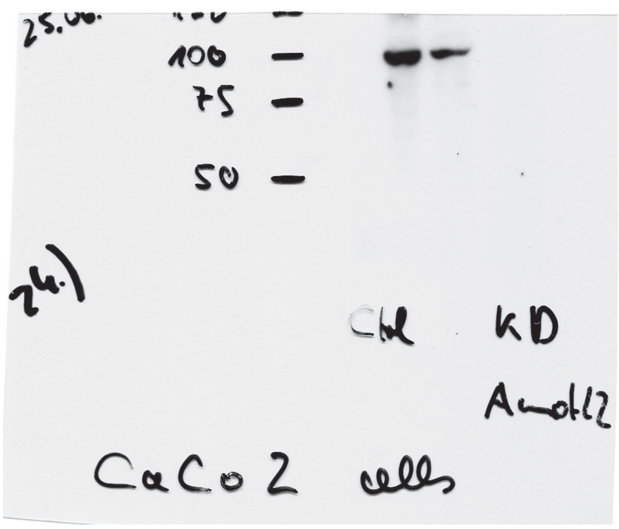

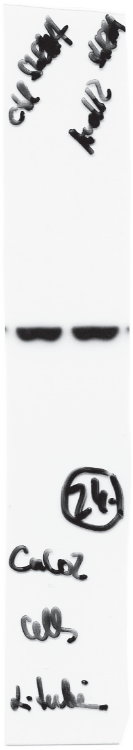

Full Full blot to supplementary Figure 2b

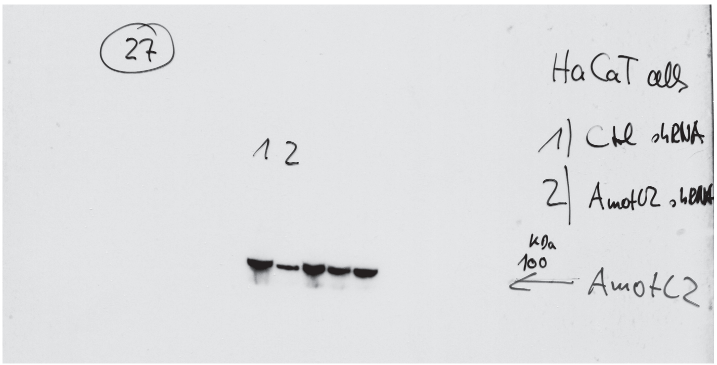

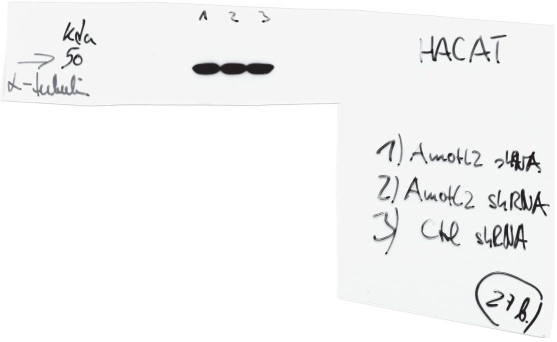

Supplement: Supplementary file 1 — The E-cadherin/AmotL2 complex organizes actin filaments required for epithelial hexagonal packing and blastocyst hatching [file 41598_2017_10102_MOESM1_ESM.pdf]
